# Capnellenes from *Capnella imbricata*: Deciphering Their Anti-Inflammatory-Associated Chemical Features

**DOI:** 10.3390/ph16070916

**Published:** 2023-06-22

**Authors:** Kuei-Hung Lai, Yu-Chen Fan, Bo-Rong Peng, Zhi-Hong Wen, Hsu-Ming Chung

**Affiliations:** 1 Graduate Institute of Pharmacognosy, College of Pharmacy, Taipei Medical University, Taipei 110301, Taiwan; kueihunglai@tmu.edu.tw (K.-H.L.); peng_br@tmu.edu.tw (B.-R.P.); 2 PhD Program in Clinical Drug Development of Herbal Medicine, College of Pharmacy, Taipei Medical University, Taipei 110301, Taiwan; 3 Traditional Herbal Medicine Research Center, Taipei Medical University Hospital, Taipei 110301, Taiwan; 4 Department of Applied Chemistry, National Pingtung University, Pingtung 900393, Taiwan; fyozhen@gmail.com; 5 Department of Marine Biotechnology and Resources, National Sun Yat-sen University, Kaohsiung 804201, Taiwan; wzh@mail.nsysu.edu.tw; 6 Institute of BioPharmaceutical Sciences, National Sun Yat-sen University, Kaohsiung 804201, Taiwan

**Keywords:** capnellene, anti-inflammation, iNOS, COX-2, ChemGPS-NP

## Abstract

Through our ongoing research on investigating new anti-inflammatory terpenoids derived from soft corals, seven capnellenes sourced from *Capnella imbricata* were discovered. Among these, three were previously unknown compounds named Δ^9(12)^-capnellene-6α,8β-diol (**1**), Δ^9(12)^-capnellene-6α,8β,10α-triol (**2**), and Δ^9(12)^-capnellene-2β,8β,10α-triol (**3**). The structures of all compounds were determined by spectroscopic analysis (IR, MS, 1D-, and 2D-NMR) and a comparison with the existing literature data. The compounds **1** and **2** were found to be the first-ever identified 6-hydroxy capnellenes. In the inflammation inhibitory assessments, compounds **1**–**7** were tested for their in vitro activities against inducible nitric oxide synthase (iNOS) and cyclooxygenase-2 (COX-2) protein expressions in LPS-induced RAW264.7 cells. Capnellenes **2** and **5** demonstrated significant reductions in iNOS levels (27.73% and 47.61%) at a concentration of 10 μM. Additionally, capnellenes **1**, **5**, and **7** (at 10 μM) exhibited statistically significant inhibitions (ranging from 7.64% to 12.57%) against COX-2 protein expressions. Our findings indicated that the oxygen-bearing functionalities at C-8 and C-10 play critical roles in inhibiting iNOS protein induction, which can promote inflammation in LPS-induced RAW264.7 cells. Furthermore, a principal component analysis tool, the chemical global positioning system for natural products (ChemGPS-NP), was applied to confirm these capnellane-based sesquiterpenes as promising candidates for future anti-inflammatory agents targeting iNOS-related targets.

## 1. Introduction

*Capnella imbricata* is a species of soft coral found in the shallow waters of the tropical and subtropical regions of the world’s oceans [1]. This soft coral has been found to produce a wide range of terpenoids, including capnellenes, which are known to possess potent anti-inflammatory properties [2,3,4].

Capnellenes are a group of terpenoid compounds that have been identified in various soft corals [2,5]. These compounds are structurally diverse, but they all contain a characteristic bicyclo[4.4.0]decane ring system. Recent research has shown that capnellenes derived from *C. imbricata* possess potent anti-inflammatory properties [3]. In particular, these compounds have been found to inhibit the production of pro-inflammatory cytokines and chemokines, which play a crucial role in the pathogenesis of inflammatory diseases [2]. The anti-inflammatory effects of capnellenes have been investigated in various in vitro and in vivo models. For example, one study found that capnellenes isolated from *C. imbricata* were able to impair vascular growth due to an imbalance of redox homeostasis [6]. It also showed that capnellenes were effective in reducing the neuroinflammatory response and were nociceptive in a neuropathic rat model [7]. Ongoing research is being conducted to explore the potential therapeutic uses of capnellenes. Additionally, the discovery of bioactive capnellenes with unique chemical characteristics is of interest in the field of marine drug development.

**Figure 1:**
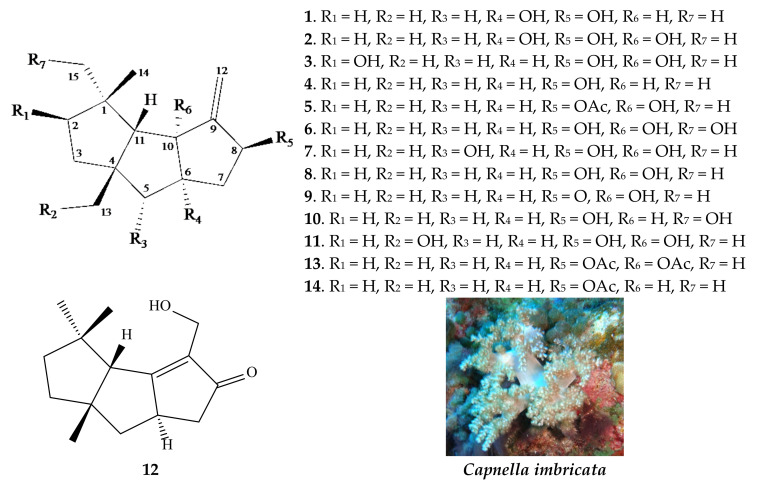
Structures of compounds **1**–**14** as well as a picture of soft coral *Capnella imbricata*.

In our current study, we investigated a series of capnellenes (**1**–**14**, Figure 1) and evaluated their potential as anti-inflammatory agents. Specifically, we assessed their effects on the expression of two pro-inflammatory proteins, iNOS (inducible nitric oxide synthase) and COX-2 (cyclooxygenase-2), in RAW264.7 macrophagic cells stimulated with lipo-polysaccharide (LPS). To gain insight into the structural–bioactivity relationship of these capnellenes, we utilized a tool called the chemical global positioning system for natural products (ChemGPS-NP), which is based on principal component analysis (PCA). This analysis allowed us to explore the relationship between the chemical structures of the capnellenes and their anti-inflammatory properties. Furthermore, to delve deeper into the anti-inflammatory potential of capnellenes targeting iNOS, we employed a molecular modeling approach. This approach enabled us to investigate the interactions between the capnellenes and the active site of iNOS, providing valuable information about their mechanism of action. Overall, our study aimed to assess the anti-inflammatory activity of capnellenes against iNOS and COX-2, explore the structural–bioactivity relationship, and gain insight into their mode of action using molecular modeling.

## 2. Results

The soft coral *C. imbricata*, collected off Taiwan’s Orchid Island, was frozen immediately after collection and then subjected to freeze-drying and powdering. After the dried powder was obtained, it underwent extraction with ethyl acetate (EtOAc) to generate an extract. This extract was then subjected to column chromatography utilizing silica gel, followed by separation using high-performance liquid chromatography (HPLC), resulting in the isolation of compounds **1**–**7**.

By analyzing the ^1^H and ^13^C NMR spectra of compounds **4**–**7**, it was determined that these molecules belonged to the capnellane-type sesquiterpenoids. The identification was made based on the spectroscopic data and a comparison with the literature references. Specifically, compounds were recognized as capnellene-8β-ol (**4**) [8], 8α-acetoxy-Δ^9(12)^-capnellene-10α-ol (**5**) [9], Δ^9(12)^-capnellene-8β,10α,15-triol (**6**) [3], and Δ^9(12)^-capnellene-5α,8β,10α-triol (**7**) [3]. Additional details and corresponding figures are available in the Supplementary Materials (Figures S26–S33).

### 2.1. Structure Determination of Compounds **1**−**3**

Δ^9(12)^-Capnellene-6α,8β-diol (**1**) was acquired in the form of an unstructured powder and exhibited a molecular formula of C_15_H_24_O_2_, as determined by high-resolution electrospray ionization mass spectrometry (HRESIMS) at *m*/*z* 259.16672 (calcd. for C_15_H_24_O_2_ + Na, 259.16685). The compound displayed an index of hydrogen deficiency (IHD) of 4. The IR spectrum showed absorption bands attributed to hydroxy (3472 cm^−1^) groups. The ^13^C NMR and HMQC spectra revealed that compound **1** had 15 carbons (Table 1), including three methyls, four sp^3^ methylenes, one sp^2^ methylene, three sp^3^ methines (including one oxymethine), three sp^3^ quaternary carbons (including an oxygenated quaternary carbon), and one quaternary sp^2^ carbon. The ^1^H NMR spectrum revealed distinct peaks corresponding to three methyl groups, five aliphatic methylene groups, and three methine groups, as indicated in Table 1. Therefore, according to the aforementioned data, four indexes of hydrogen deficiency were accounted for, and compound **1** was identified as having a double bond and three rings.

**Figure 2:**
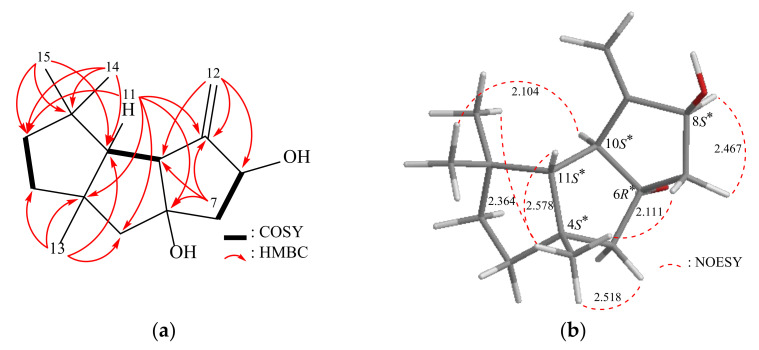
(**a**) The ^1^H–^1^H COSY and selective HMBC correlations and (**b**) stereoview of **1** and the calculated distances (Å) between selected protons having key NOESY correlations.

By conducting a ^1^H–^1^H correlation spectroscopy (COSY) experiment on compound **1**, we were able to determine the proton connectivity between H_2_-2 and H_2_-3, H_2_-7 and H-8, as well as H-10 and H-11 (as shown in Figure 2). The heteronuclear multiple-bond coherence (HMBC) experiment provided valuable assistance in establishing these spin systems. The significant long-range correlations between protons and quaternary carbons in the molecule were observed, including H-3, H-10, H_3_-14, and H_2_-15 with C-1; H-2, H-3, H_2_-5, H-11, and H_3_-13 with C-4; H-5β, H-7β, and H-11 with C-6; and H-7β, H-11, and H_2_-12 with C-9, with coupling constants of ^2^*J* or ^3^*J*. The presence of tertiary methyl groups at C-1 and C-4 was verified through HMBC correlations, specifically between H_3_-13 and C-3, C-4, C-5, and C-11; H_3_-14 and C-1, C-2, C-11, C-15; and H_3_-15 and C-1, C-2, C-11, C-14. The presence of an exocyclic double bond at C-8 was confirmed through HMBC correlations, specifically between H_2_-12 and C-8, C-9, and C-10. By considering these data, along with the HMBC correlations observed between H-10 and C-1, C-11, and H-11 and C-2, C-4, C-5, C-6, C-9, C-10, C-13, C-14, C-15, the main carbon skeleton of compound **1** was successfully determined.

The relative configuration of compound **1** was determined based on the interactions observed in the nuclear Overhauser effect spectroscopy (NOESY) data (Figure 2). Assuming the β-orientation of H-11 (*δ*_H_ 1.62, m), the NOESY correlations from H-11/H_3_-13 (*δ*_H_ 1.21, s) and H_3_-13/H_3_-14, H-5β (*δ*_H_ 1.79, d, *J* = 13.5 Hz), H-7β (*δ*_H_ 1.83, dd, *J* = 13.0, 10.0 Hz) confirmed that these protons were on the same face. Additionally, further correlations from H-10 (*δ*_H_ 2.68, dd, *J* = 7.0, 2.0 Hz)/H_3_-15 (*δ*_H_ 1.10, s) and from H-8 (*δ*_H_ 4.80, br s)/H-7α (*δ*_H_ 2.26, dd, *J* = 13.0, 7.5 Hz) suggested that all of these groups should be situated on the opposing side of H-11. From the above observations, the 4S*, 6R*, 8S*, 10S* and 11S* relative configurations of **1** were established (Supplementary Materials, Figures S1–S8).

To determine the absolute stereochemistry of compound **1**, Gaussian 16 software was utilized to calculate the conformation, optimize the structure, and determine the specific optional rotation (SOR) value. Possible configurations of **1**-4*S*,6*S*,8*S*,10*S*,11*S* and **1**-4*R*,6*R*,8*R*,10*R*,11*R* were input into the software, and the SOR values were obtained (Table 2). The calculated SOR value for **1**-4*S*,6*S*,8*S*,10*S*,11*S* (+50) aligned with the experimental result of **1** (positive). Based on these findings, the configurations of the stereogenic centers in compound 1 were identified as (4*S*,6*S*,8*S*,10*S*,11*S*). Consequently, the structure of compound **1** was successfully determined.

**Figure 3:**
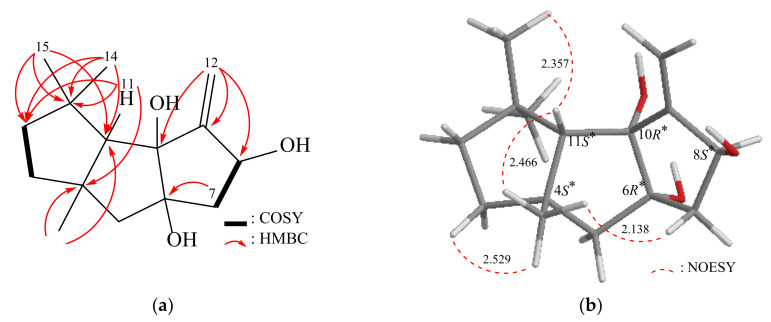
(**a**) The ^1^H–^1^H COSY and selective HMBC correlations and (**b**) stereoview of **2** and the calculated distances (Å) between selected protons having key NOESY correlations.

Obtained in the form of an amorphous powder, Δ^9(12)^-Capnellene-6α,8β,10α-triol (**2**) exhibited a molecular formula of C_15_H_24_O_3_ as determined by HRESIMS, with an observed *m*/*z* of 275.16147 (calcd. for C_15_H_24_O_3_ + Na, 275.16177). The compound displayed an IHD value of 4. The IR spectrum exhibited absorption bands at 3472 cm^−1^, which were assigned to the presence of hydroxy groups. The ^13^C NMR and HMQC spectra provided information about the carbon atoms in compound **2**, indicating the presence of 15 carbons (Table 3). These include three methyl groups, four sp^3^ methylene groups, one sp^2^ methylene group, two sp^3^ methine groups (including one oxymethine), four sp^3^ quaternary carbons (including two oxygenated quaternary carbons), and one quaternary sp^2^ carbon. The ^1^H NMR spectrum exhibited distinct signals corresponding to three singlet methyl groups, five aliphatic methylene groups, and two methine groups, all of which were observed and recorded in Table 3. A comparison of the MS spectral data revealed that compound **2** exhibited an additional oxygen atom in comparison to compound **1**. Nevertheless, the analysis of the ^1^H and ^13^C-NMR spectra indicated that the signals attributed to the C-10 methine in **2** vanished and were substituted by signals characteristic of an additional oxygenated quaternary carbon. By analyzing the 2D NMR spectra (COSY and HMBC) of **2**, the previously mentioned elucidation was confirmed, thereby establishing the planar structure (Figure 3). According to the NOESY spectrum (Figure 3), the 4S*, 6R*, 8S*, 10R*, and 11S* relative configurations of **2** were established (Supplementary Materials, Figures S9–S16). Compound **2**’s absolute stereochemistry was determined using Gaussian 16 software. Possible configurations of **2**-4*S*,6*S*,8*S*,10*S*,11*S* and **2**-4*R*,6*R*,8*R*,10*R*,11*R* were inputted, and SOR values were obtained (Table 2). The calculated SOR value for **2**-4*S*,6*S*,8*S*,10*S*,11*S* (+42) matched the experimental result of **2** (positive). These findings confirmed the configurations of the stereogenic centers in compound **2** as (4*S*,6*S*,8*S*,10*S*,11*S*).

**Figure 4:**
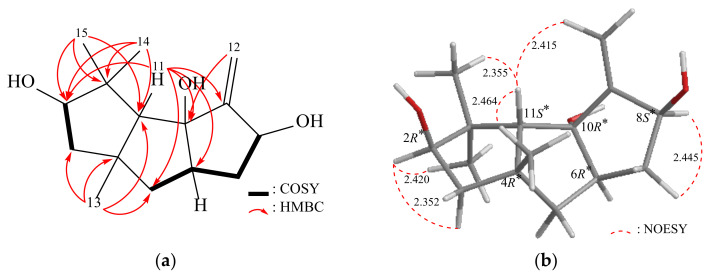
(**a**) The ^1^H–^1^H COSY and selective HMBC correlations and (**b**) stereoview of **3** and the calculated distances (Å) between selected protons having key NOESY correlations.

Δ^9(12)^-Capnellene-2β,8β,10α-triol (**3**) was obtained as an amorphous powder, with a molecular formula C_15_H_24_O_3_ determined by HRESIMS at *m*/*z* 275.16144 (calcd. for C_15_H_24_O_3_ + Na, 275.16177) (IHD = 4). The IR spectrum exhibited absorption bands indicative of hydroxy (3548 cm^−1^) groups. Analysis of the ^13^C NMR, DEPT, and HMQC spectra revealed that compound **3** consisted of 15 carbons (Table 4), including three methyls, three sp^3^ methylenes, one sp^2^ methylene, four sp^3^ methines (including two oxymethines), three sp^3^ quaternary carbons (including an oxygenated quaternary carbon), and one quaternary sp^2^ carbon. The ^1^H NMR spectrum displayed signals corresponding to three singlet methyls, five aliphatic methylenes, and two methines (Table 4). The similarity between the ^1^H NMR data of **3** and Δ^9(12)^-Capnellene-2ξ,8β,10α-triol suggested a close structural resemblance [2]. The interpretation of the 2D NMR spectra (COSY and HMBC) of **3** confirmed the aforementioned elucidation and established the planar structure (Figure 4). The β-configurations of the hydroxy groups at C-2 were assigned primarily based on NOESY correlations between H-11/H2-12, H3-13, H3-14, and H-2/H3-15 (Figure 4). Consequently, the stereogenic centers of **3** were determined as 2R*, 4R*, 6R*, 8S*, 10R*, and 11S* (Supplementary Materials, Figures S17–S25). Likewise, during the Gaussian calculation, the calculated SOR value for **3**-2*R*,4*S*,6*S*,8*S*,10*R*,11*R* (+50) aligned with the experimental result of **3** (positive) (Table 2). These results confirmed that the stereogenic centers in compound **3** have the configurations of (2*R*,4*S*,6*S*,8*S*,10*R*,11*R*).

### 2.2. Anti-Inflammatory Activity of the Isolated Capnellenes

In an in vitro anti-inflammatory activity assay, the expression levels of pro-inflammatory iNOS and COX-2 proteins were evaluated using Western blot analysis in LPS-stimulated RAW264.7 macrophage cells. (Figure S30 and Table 5). Capnellenes **2** and **5** were observed to significantly decrease the levels of iNOS (27.73% and 47.61%) without causing any cytotoxicity (data not shown), when administered at a concentration of 10 μM, as compared to the control cells that were stimulated with LPS only. Furthermore, capnellenes **1**, **5**, and **7** (at 10 μM) showed statistically significant inhibitions (ranging from 7.64–12.57%) against COX-2 protein expressions. It is worth noting that compounds **2** and **5** exhibited the inhibitory activity against iNOS but did not have any impact on COX-2. On the other hand, compound **1** only affected COX-2 but not iNOS. Reviewing our previous reported results [2], the strong iNOS inhibitory properties of capnellenes were indicated, in particular, compounds **8** (98.8%) and **9** (65.2%). Based on the above results, the important roles of both 8- or 10-oxygenated functionalities were deduced. Any other oxygenated groups attaching to C-2, C-5, C-6, C-13, and C-15 de-creased the anti-inflammatory activity against iNOS.

### 2.3. ChemGPS-NP-Based Analysis of the Anti-Inflammatory Capnellenes

The Backlund group introduced ChemGPS-NP, a model based on principle component analysis (PCA) for analyzing natural products in 2007 [10,11]. They subsequently established an online system in 2009 that offers the analysis of eight principle components (PCs). Over the past few years, this computational tool has been utilized to study structure–activity relationships (SAR) [12,13,14,15,16] and to investigate potential pharmacological targets that can guide drug discovery using natural products [17,18].

**Figure 5:**
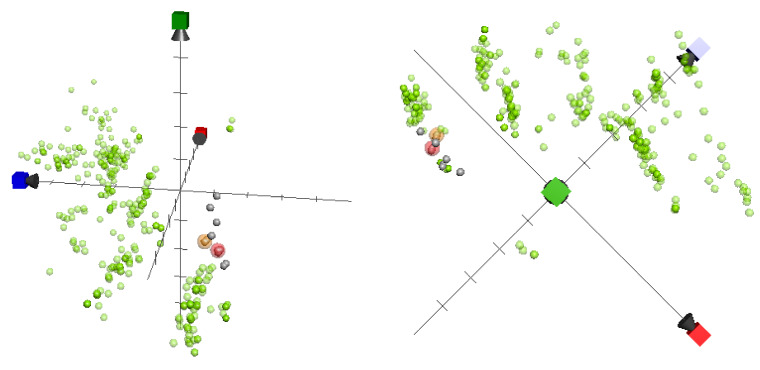
ChemGPS-NP analysis of the chemical space plotted for capnellene-type sesquiterpenes reported by our lab (grey: non-active; red transparent sphere: inhibition rate > 90%; and yellow transparent sphere: inhibition rate > 50%, at 10 μM against iNOS expression), as well as previously studied 342 iNOS inhibitors (light green) sorted from the database of ChEMBL. The three-dimensional score plot was generated using the principle components, where PC1 (red) describes properties related to size, shape, and polarizability; PC2 (blue) represents properties related to aromatics and conjugation; and PC3 (green) describes properties related to lipophilicity, polarity, and H-bond capacity.

In this study, we utilized ChemGPS-NP to establish the chemical relationships between capnellenes discussed in our present research and those mentioned in our previous publications [2] that possess anti-inflammatory activity. We mapped a total of 14 capnellene sesquiterpenes (**1**–**14**) in a chemical space using PC score predictions from the online tool ChemGPS-NPWeb (Figure 5). Additionally, we retrieved previously disclosed inhibitors that target inflammation-related proteins iNOS from the ChEMBL online database and included them in the analysis. These data were also included in analyzing the graphics. The active capnellenes particularly explored the negative quadrant of the third dimension (PC3, which describes lipophilicity, polarity, and H-bond capacity) and were located in close proximity to the group of iNOS inhibitors that were previously identified. These findings provide further evidence supporting the crucial role of oxygenated functionalities in the anti-inflammatory properties of capnellene compounds that target iNOS.

**Figure 6:**
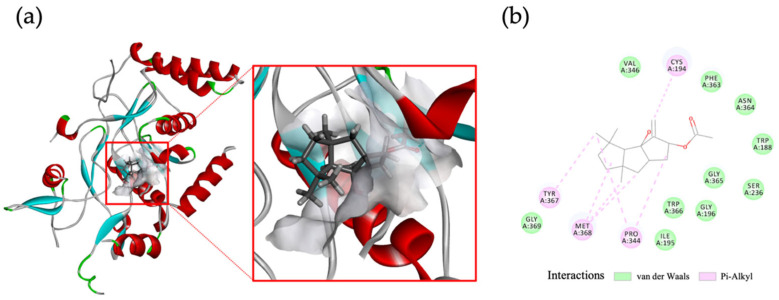
Molecular docking of compound **5** within the murine iNOS oxygenase complex. compound **5** was simulated to exhibit interaction with M368, P344, and other amino acids of iNOS: (**a**) predicted binding pose of **5**; (**b**) 2D diagram of ligand–protein interaction. Crystal structure of murine iNOS oxygenase complex (PDB: 2ORO). iNOS protein, compound **5** molecule, and active site are, respectively, shown in blue/red, dark grey stick, and light grey.

The intriguing discovery in our study was that compound **5**, which exhibited the highest activity against iNOS, did not have any impact on COX-2, indicating the specific targeting of capnellenes toward iNOS protein. To investigate further, we conducted molecular docking analysis to explore whether compound **5** affects the active site of the iNOS oxygenase complex. The docking results revealed interactions between **5** and amino acids such as M368 and P344, among others (CDOCKER interaction energy: −33.19 kcal/mol), as depicted in Figure 6. These findings provide additional support for the anti-inflammatory efficacy of compound **5** through its targeted action on iNOS. 

## 3. Discussion

Our current study discusses the investigation of capnellenes, a group of terpenoid compounds produced by the soft coral *Capnella imbricata*, for their potential as anti-inflammatory agents. The researchers isolated and characterized several capnellenes from the coral and evaluated their effects on pro-inflammatory proteins iNOS and COX-2 in macrophage cells stimulated with lipopolysaccharide (LPS).

The study employed various techniques to gain insights into the structural–bioactivity relationship and the mode of action of capnellenes. Chemical analysis using NMR and mass spectrometry allowed the identification and characterization of the isolated compounds. We determined the relative configurations of the capnellenes through spectroscopic techniques and NOESY analysis. 

In the anti-inflammatory activity assay, capnellenes **2** and **5** exhibited significant decreases in iNOS levels without cytotoxicity when administered at a concentration of 10 μM. Furthermore, capnellenes **1**, **5**, and **7** showed statistically significant inhibitions against COX-2 protein expressions. To explore the structural-bioactivity relationship, the researchers employed a tool called ChemGPS-NP, based on PCA, to analyze the chemical space of the capnellenes. This analysis provided insights into the chemical relationships between the capnellenes and their anti-inflammatory properties. In addition, the study used molecular modeling techniques to investigate the interactions between capnellenes and the active site of iNOS, providing valuable information about their mechanism of action.

It is worth noting that previous studies have also investigated the anti-inflammatory properties of capnellenes. For instance, studies isolated and characterized capnellenes from *Capnella imbricata* collected from Taiwan, and assessed their anti-inflammatory activity by examining their effects on the release of elastase and generation of superoxide anions by human neutrophils [3]. These studies provided additional evidence supporting the anti-inflammatory potential of capnellenes. Moreover, a study explored the anti-neuroinflammatory properties of capnellenes GB9 and GB10 using in vitro and in vivo models [7]. Their findings demonstrated that capnellenes exhibited anti-neuroinflammatory and anti-nociceptive properties in microglial cells and neuropathic rat models, highlighting their potential for the treatment of neuroinflammatory diseases. Interestingly, another study by Lin et al. (2023) investigated the vascular impacts of capnellenes, specifically GB9, in zebrafish embryos [6]. The researchers observed that GB9 treatment impaired vascular development, including intersegmental vessel (ISV) growth and caudal vein plexus (CVP) patterning, indicating a potential influence of capnellenes on embryonic vascular development. 

In conclusion, the present study contributes to the understanding of capnellenes as potential anti-inflammatory agents derived from *Capnella imbricata*. The combination of chemical analysis, bioassays, and molecular modeling techniques shed light on the structural–bioactivity relationship and mode of action of capnellenes. Further research in the field of marine drug development is warranted to explore the therapeutic potential of capnellenes and their derivatives in the treatment of inflammatory diseases.

## 4. Materials and Methods

### 4.1. General Procedures

The Jasco P-1010 digital polarimeter (JASCO Corporation, Tokyo, Japan) was used to measure the optical rotations of the isolates. IR spectra were obtained using the Nicolet iS5 FT-IR spectrophotometer (Thermo Fisher Scientific, Waltham, MA, USA). NMR spectra were acquired using either a Varian Unity INOVA500 FT-NMR (Varian Inc., Palo Alto, CA, USA) or Jeol ECZ NMR spectrometer (Jeol, Tokyo, Japan), operating at 500 MHz (or 600 MHz) for ^1^H and 125 MHz (or 150 MHz) for ^13^C, in CDCl_3_. The residual CHCl_3_ signal (δH 7.26 ppm) and CDCl_3_ (δC 77.1 ppm) were used as the internal standard for ^1^H and ^13^C NMR, respectively, with coupling constants (*J*) reported in Hz. Bruker 7 Tesla SolariX FTMS mass spectrometer (Bruker, Bremen, Germany) was used to obtain ESIMS and HRESIMS data. Column chromatography (CC) with silica gel (particle size, 230–400 mesh; Merck, Darmstadt, Germany) was employed to separate the extracted samples. Thin-layer chromatography (TLC) plates precoated with silica gel (Kieselgel 60 F 254, Merck, Darmstadt, Germany) were utilized, and for visualization, the TLC plates were sprayed with 10% (*v*/*v*) aqueous sulfuric acid solution and then heated at 105 °C until spots were observed. For HPLC, a Hitachi L-7100 pump and a Rheodyne 7725 injection port (Hitachi, Tokyo, Japan) were used, and a semi-preparative normal phase column (Hibar 250 × 10 mm, Supelco, silica gel 60, 5 μm) (Merck, Darmstadt, Germany) was employed.

### 4.2. Animal Material

In June 2017, specimens of the octocoral *C. imbricata* were manually collected using self-contained underwater breathing apparatus (SCUBA) from the coast of Orchid Island (Lanyu Island). The samples were then kept in a freezer at a temperature of −20 °C until extraction. A voucher specimen bearing the number NMMBA-TW-SI-2017-030 was stored in the National Museum of Marine Biology and Aquarium, Taiwan.

### 4.3. Extraction and Isolation

*Capnella imbricata* (1287.6 g fresh weight) was collected and freeze-dried. The organism material (265.3 g dry weight) was minced and extracted exhaustively with EtOAc (2L × 5). The EtOAc extract was evaporated to yield a residue (4.98 g), which was subjected to open CC on silica gel eluting with an *n*-hexane/EtOAc/acetone/methanol (from 100% *n*-hexane to 100% methanol) to present 18 fractions (fractions 1−18). Fraction 12 was chromatographed with Si C.C. using *n*-hexane/acetone (4:1) to obtain fractions 12A–12I. Fraction 12B was separated by NP-HPLC using a mixture of *n*-hexane/acetone (5:1) to yield Δ^9(12)^-capnellene-6α,8β-diol (**1**) (0.3 mg) and Δ^9(12)^-capnellene-6α,8β,10α-triol (**2**) (0.8 mg). Fraction 13 was chromatographed using silica gel CC using *n*-hexane/acetone (3:1) to yield Δ^9(12)^-capnellene-2β,8β,10α-diol (**3**) (6.9 mg). Fraction 6 was chromatographed using silica gel CC using *n*-hexane/EtOAc/acetone (from *n*-hexane/EtOAc (50:1) to 100% acetone) to yield capnellene-8β-ol (**4**) (60.5 mg) and 8α-acetoxy-Δ^9(12)^-capnellene-10α-ol (**5**) (35.8 mg). Using a solvent mixture (H/A = 5/1), fraction 14 underwent additional separation via NP-HPLC, resulting in the isolation of 1.5 mg of Δ^9(12)^-capnellene-8β,10α,15-triol (**6**) [3] and 2.2 mg of Δ^9(12)^-capnellene-5α,8β,10α-triol (**7**) [3].

Δ^9(12)^-Capnellene-6α,8β-diol (**1**): Amorphous powder; ${\left[\alpha \right]}_{D}^{25}$ +49 (*c* 0.15, CHCl_3_); IR (KBr) *ν*_max_ 3472, 2932 and 2866 cm^−1^; ^1^H (500 MHz, CDCl_3_) and ^13^C (125 MHz, CDCl_3_) data: Table 1; ESIMS *m*/*z* 259 [M + Na]^+^; HRESIMS *m*/*z* 259.16672 ([M + Na]^+^, calcd for C_15_H_24_O_2_ + Na, 259.16685).

Δ^9(12)^-Capnellene-6α,8β,10α-triol (**2**): Amorphous powder; ${\left[\alpha \right]}_{D}^{25}$ +2.99 (*c* 3.01, CHCl_3_); IR (KBr) *ν*_max_ 3465, 2929 and 2867 cm^−1^; ^1^H (500 MHz, CDCl_3_) and ^13^C (125 MHz, CDCl_3_) data: Table 2; ESIMS *m*/*z* 275 [M + Na]^+^; HRESIMS *m*/*z* 275.16147 ([M + Na]^+^, calcd for C_15_H_24_O_3_ + Na, 275.16177).

Δ^9(12)^-Capnellene-2β,8β,10α-triol (**3**): Amorphous yellow powder; ${\left[\alpha \right]}_{D}^{25}$ +23 (*c* 0.35, CHCl_3_); IR (KBr) *ν*_max_ 3429 and 2948 cm^−1^; ^1^H (600 MHz, CDCl_3_) and ^13^C (150 MHz, CDCl_3_) data: Table 3; ESIMS *m*/*z* 275 [M + Na]^+^; HRESIMS *m*/*z* 275.16144 ([M + Na]^+^, calcd for C_15_H_24_O_3_ + Na, 275.16177).

Capnellene-8β-ol (**4**): Amorphous powder; ${\left[\alpha \right]}_{D}^{25}$ +0.36 (*c* 30.0, CHCl_3_); IR (KBr) *ν*_max_ 3492, 2940 and 2864 cm^−1^; ^1^H and ^13^C NMR data were found to be in similar with previous studies [8]; ESIMS *m*/*z* 242 [M + Na]^+^.

8α-Acetoxy-Δ^9(12)^-capnellene-10α-ol (**5**): Amorphous yellow powder; ${\left[\alpha \right]}_{D}^{25}$ +3.96 (*c* 1.79, CHCl_3_); IR (KBr) *ν*_max_ 3503, 2930, 2864, and 1735 cm^−1^; ^1^H and ^13^C NMR data were found to be similar with previous studies [9]; ESIMS *m*/*z* 301 [M + Na]^+^.

Δ^9(12)^-Capnellene-8β,10α,15-triol (**6**): Amorphous powder; ${\left[\alpha \right]}_{D}^{25}$ +0.41 (*c* 0.1, CHCl_3_); IR (KBr) *ν*_max_ 3250 cm^−1^; ^1^H (600 MHz, CDCl_3_) and ^13^C (150 MHz, CDCl_3_) data: Table 1; ESIMS *m*/*z* 275 [M + Na]^+^; HRESIMS *m*/*z* 275.16191 ([M + Na]^+^, calcd. for C_15_H_24_O_3_ + Na, 275.16177).

Δ^9(12)^-Capnellene-5α,8β,10α-triol (**7**): Amorphous powder; ${\left[\alpha \right]}_{D}^{25}$ −1.47 (*c* 0.1, CHCl_3_); IR (KBr) *ν*_max_ 3379 cm^−1^; ^1^H and ^13^C NMR data (Supplementary Materials, Figures S10–S15) were found to be in similar with previous studies [7]; ESIMS *m*/*z* 275 [M + Na]^+^.

### 4.4. In Silico Calculations

The molecular structures were subjected to in silico calculations to optimize their energy at the MM2 level. The resulting mol file was then utilized in GaussView 6.1 (Gaussian Inc.; Wallingford, CT, USA) with the MMFF94 force field and the GMMX package to perform a conformational search. The obtained data were imported into Gaussian 16 software (Gaussian Inc.; Wallingford, CT, USA) for further optimization using the time-dependent density functional theory (TDDFT) methodology at the B3LYP/6-311(d,p) levels, considering the solvent phase for SOR calculation. The computed SOR results were subsequently averaged based on the proportion of each conformer.

### 4.5. In Vitro Anti-Inflammatory Test

The RAW264.7 macrophage cell line was obtained from ATCC. The ability of compounds **1**–**7** to inhibit the expression of iNOS (inducible nitric oxide synthetase) and COX-2 (cyclooxygenase-2) pro-inflammatory proteins in LPS-induced RAW264.7 macrophage cells was assessed to determine their in vitro anti-inflammatory activities. A detailed description of the method used for this evaluation was provided in previous publications [2].

### 4.6. ChemGPS-NP Analysis

ChemGPS-NP is a navigation tool for exploring the biologically relevant chemical space that utilizes 35 carefully selected chemical descriptors to describe physical–chemical properties such as shape, size, polarizability, polarity, flexibility, lipophilicity, hydrogen bond capacity, and rigidity. This tool consists of eight principal components (PCs) that are used to make predictions based on the structural information of compounds provided as simplified molecular input line entry specifications (SMILES) derived from ChemBioDraw version 16.0. To map all the briarane-type diterpenoids into the ChemGPS-NP chemical property space, we used the online tool ChemGPS-NPWeb (http://chemgps.bmc.uu.se, accessed on 17 March 2023) and Grapher 2.6 software (Mac OS). Additionally, we included previously studied iNOS and COX-2 inhibitors from the database of ChEMBL in the analysis. To select active agents, we sorted the data based on the definition of IC_50_ < 1000 nM provided by ChEMBL.

### 4.7. Molecular Docking

The analysis of ligand–protein interactions was conducted using in silico docking analysis performed with Discovery Studio 2021 (Biovia, Corp., San Diego, CA, USA). The 3D structure of compound **5** was simulated using ChemDraw. The crystal structure of the murine iNOS oxygenase complex, obtained from the RCSB Protein Data Bank (PDB ID: 2ORO), was utilized. Initial protein structures were generated using the CHARMm force field and subsequently minimized. The active site was defined by setting a sphere radius. Docking poses were generated following the CDOCKER protocol using Discovery Studio software.

## 5. Conclusions

In conclusion, this study investigated new anti-inflammatory terpenoids derived from soft corals and discovered seven capnellenes sourced from *Capnella imbricata*, including three previously unknown compounds (**1**–**3**). The study also found that compounds **1** and **2** were the first-ever identified 6-hydroxy capnellenes. In vitro tests against iNOS and COX-2 protein expressions showed that the oxygen-bearing functionalities at C-8 and C-10 play critical roles in inhibiting iNOS protein induction. Furthermore, the ChemGPS-NP tool confirmed these capnellane-based sesquiterpenes as promising candidates for future anti-inflammatory agents targeting iNOS-related targets. These findings provide new insights into the potential development of anti-inflammatory agents from marine-derived leads.

## Figures and Tables

**Table 1:** ^1^H and ^13^C NMR data for compound **1**.

C/H	*δ*_H_ (*J* in Hz) ^a^	*δ*_C_ ^b^, Type ^c^
1		42.1, C
2	1.47 m	40.6, CH_2_
3	1.64 m	41.3, CH_2_
4		49.8, C
5α/β	2.04 d (13.5); 1.79 d (13.5)	55.4 CH_2_
6		88.0, C
7α/β	2.26 dd (13.0, 7.5); 1.83 dd (13.0, 10.0)	48.3, CH_2_
8	4.80 br s	75.1, CH
9		159.7, C
10	2.68 dd (7.0, 2.0)	58.3, CH
11	1.62 m	69.1, CH
12a/b	4.99 t (2.0); 5.17 t (2.0)	107.8, CH_2_
13	1.21 s	32.1, CH_3_
14	1.04 s	30.0, CH_3_
15	1.10 s	26.6, CH_3_

^a^ Spectra recorded at 500 MHz in CDCl_3_ at 25 °C. ^b^ Spectra recorded at 125 MHz in CDCl_3_ at 25 °C. ^c^ Multiplicity deduced by HMQC spectra.

**Table 2:** Experimental and calculated specific optical rotation values of **1**–**3**.

	Cald. Value ^a^	Exp. Value
Exp. **1** ^b^		49
Cald. **1**-4*S*,6*S*,8*S*,10*S*,11*S*	50	
Cald. **1**-4*R*,6*R*,8*R*,10*R*,11*R*	−50	
Exp. **2** ^c^		2.99
Cald. **2**-4*S*,6*S*,8*S*,10*R*,11*S*	42	
Cald. **2**-4*R*,6*R*,8*R*,10*S*,11*R*	−42	
Exp. **3** ^d^		23
Cald. **3**-2*R*,4*S*,6*S*,8*S*,10*R*,11*R*	50	
Cald. **3**-2*S*,4*R*,6*R*,8*R*,10*S*,11*S*	−50	

^a^ Solvent phase in CHCl_3_; ^b^ ${\left[\alpha \right]}_{D}^{25}$ (c 0.15, CHCl_3_); ^c^ ${\left[\alpha \right]}_{D}^{25}$ (c 3.01, CHCl_3_); ^d^ ${\left[\alpha \right]}_{D}^{25}$ (c 0.35, CHCl_3_).

**Table 3:** ^1^H and ^13^C NMR data for compound **2**.

C/H	*δ*_H_ (*J* in Hz) ^a^	*δ*_C_ ^b^, Type ^c^
1		44.2, C
2a/b	1.34 m; 2.05 m	41.0, CH_2_
3α/β	1.71 dd (8.0, 2.5); 1.64 dd (8.0, 2.0)	42.4, CH_2_
4		47.7, C
5α/β	1.83 d (13.5); 1.97 d (13.5)	52.1 CH_2_
6		72.7, C
7α/β	2.33 dd (16.5, 8.0); 1.91 dd (15.0, 8.0)	43.6, CH_2_
8	4.79 br s	87.4, CH
9		160.2, C
10		89.7, C
11	1.88 s	67.0, CH
12a/b	5.38 d (2.0); 5.42 d (2.0)	112.5, CH_2_
13	1.31 s	33.4, CH_3_
14	1.03 s	31.8, CH_3_
15	1.38 s	25.0, CH_3_

^a^ Spectra recorded at 500 MHz in CDCl_3_ at 25 °C. ^b^ Spectra recorded at 125 MHz in CDCl_3_ at 25 °C. ^c^ Multiplicity deduced by HMQC spectra.

**Table 4:** ^1^H and ^13^C NMR data for compound **3** and ^1^H NMR data for Δ^9(12)^-Capnellene-2ξ,8β,10α-triol [9].

C/H	3		Δ^9(12)^-Capnellene-2ξ,8β,10α-Triol
	*δ*_H_ (*J* in Hz) ^a^	*δ*_C_ ^b^, Type ^c^	*δ*_H_ (*J* in Hz) ^d^
1		46.9, C	
2	4.03 dd (5.4, 5.4)	82.2, CH	3.70 m
3α/β	2.09 dd (13.8, 5.4); 1.55 dd (13.8, 5.4)	50.0, CH_2_	
4		47.6, C	
5a/b	1.48 m; 2.04 dd (13.8, 8.4)	46.8 CH_2_	
6	2.34 m	51.1, CH	
7α/β	2.32 m; 1.50 m	38.1, CH_2_	
8	4.74 m	73.7, CH	4.70 m
9		162.2, C	
10		90.5, C	
11	2.17 s	64.7, CH	
12a/b	5.34 d (1.8); 5.39 d (1.8)	110.3, CH_2_	5.37 m
13	1.27 s	34.4, CH_3_	1.28 s
14	1.11 s	24.2, CH_3_	1.45 s
15	1.27 s	23.0, CH_3_	1.16 s

^a^ Spectra recorded at 600 MHz in CDCl_3_ at 25 °C. ^b^ Spectra recorded at 150 MHz in CDCl_3_ at 25 °C. ^c^ Multiplicity deduced by DEPT and HMQC spectra. ^d^ Spectra recorded at 25.5 MHz in CDCl_3_.

**Table 5:** Effects of capnellenes **1**–**7** and **8**–**9** (referenced) on LPS-induced iNOS and COX-2 protein expressions in macrophages.

Compounds (10 μM)	iNOS	COX-2	*β*-actin
	Expression (% of LPS) ^c^		
vehicle	1.99 ± 0.94	1.30 ± 0.25	98.37 ± 1.75
LPS	100.00 ± 0.14	100.00 ± 0.23	103.82 ± 2.47
**1**	98.92 ± 4.23	87.02 ± 3.12	98.61 ± 3.17
**2**	72.27 ± 1.55	98.29 ± 1.52	94.51 ± 3.52
**3**	99.10 ± 4.89	101.13 ± 2.35	98.14 ± 2.49
**4**	100.75 ± 3.41	95.74 ± 1.50	97.84 ± 2.71
**5**	52.39 ± 1.49	92.36 ± 2.19	94.01 ± 3.88
**6**	98.29 ± 1.57	99.66 ± 2.50	98.56 ± 3.75
**7**	88.24 ± 2.50	89.51 ± 1.59	99.65 ± 2.88
**8** ^b^	1.20 ± 0.10	24.80 ± 7.50	-
**9** ^b^	34.80 ± 10.20	-	-
Dex ^a^	61.92 ± 5.15	28.31 ± 0.86	100.05 ± 2.86

^a^ Dexamethasone (DEX, 10 μM) was used as a positive control. ^b^ The data referenced from the literature [2]; ^c^ data were normalized to those of cells treated with LPS alone.

## Data Availability

The data presented in this study are available in the article and the Supplementary Materials.

## Supplementary Materials

The following supporting information can be downloaded at: https://www.mdpi.com/article/10.3390/ph16070916/s1, Figures S1–S33: HRESIMS, IR, and NMR spectra, Figure S34: Effects of capnellenes **1**–**7** on the protein expression levels of pro-inflammatory iNOS and COX-2.
