# Supplementary material for: Capnellenes from Capnella imbricata: Deciphering Their Anti-Inflammatory-Associated Chemical Features
Source: Pharmaceuticals (Basel). 2023 Jun 22;16(7):916. doi: 10.3390/ph16070916 (PMC10383453; doi:10.3390/ph16070916)
Supplement: Supplementary file 1 [file pharmaceuticals-16-00916-s001.zip › pharmaceuticals-2425744-supplementary.pdf]

Supplementary materials

Capnellenes from *Capnella imbricata*: Deciphering their Anti-Inflammatory-Associated Chemical Features

Kuei-Hung Lai [1, 2, 3, †], Yu-Chen Fan [4, †], Bo-Rong Peng [1], Zhi-Hong Wen [5, 6] and Hsu-Ming Chung [4, *]

[1] Graduate Institute of Pharmacognosy, College of Pharmacy, Taipei Medical University, Taipei 11031, Taiwan; kueihunglai@tmu.edu.tw
[2] PhD Program in Clinical Drug Development of Herbal Medicine, College of Pharmacy, Taipei Medical University, Taipei 11031, Taiwan
[3] Traditional Herbal Medicine Research Center, Taipei Medical University Hospital, Taipei 11031, Taiwan
[4] Department of Applied Chemistry, National Pingtung University, Pingtung 900393, Taiwan
[5] Department of Marine Biotechnology and Resources, National Sun Yat-sen University, Kaohsiung 804201, Taiwan
[6] Institute of BioPharmaceutical Sciences, National Sun Yat-sen University, Kaohsiung 804201, Taiwan
* Correspondence: shiuanmin@mail.nptu.edu.tw; Tel.: +886-8-766-3800 (ext. 33253)
† These authors contributed equally to this work.

Contents

Figure S1. HRESIMS spectrum of **1**……… 3
Figure S2. IR Spectrum of **1** in $CDCl_3$……… 3
Figure S3. ^{1}H NMR spectrum of **1** in $CDCl_3$ at 500 MHz……… 4
Figure S4. ^{13}C NMR spectrum of **1** in $CDCl_3$ at 125 MHz……… 4
Figure S5. HMQC spectrum of **1**……… 5
Figure S6. COSY spectrum of **1**……… 5
Figure S7. HMBC spectrum of **1**……… 6
Figure S8. NOESY spectrum of **1**……… 6
Figure S9. HRESIMS spectrum of **2**……… 7
Figure S10. IR Spectrum of **2** in $CDCl_3$……… 7
Figure S11. ^{1}H NMR spectrum of **2** in $CDCl_3$ at 500 MHz……… 8
Figure S12. ^{13}C NMR spectrum of **2** in $CDCl_3$ at 125 MHz……… 8
Figure S13. HMQC spectrum of **2**……… 9
Figure S14. COSY spectrum of **2**……… 9
Figure S15. HMBC spectrum of **2**……… 10
Figure S16. NOESY spectrum of **2**……… 10
Figure S17. HRESIMS spectrum of **3**……… 11
Figure S18. IR Spectrum of **3** in $CDCl_3$……… 11
Figure S19. ^{1}H NMR spectrum of **3** in $CDCl_3$ at 600 MHz……… 12
Figure S20. ^{13}C NMR spectrum of **3** in $CDCl_3$ at 150 MHz……… 12
Figure S21. DEPT spectrum of **3**……… 13
Figure S22. HMQC spectrum of **3**……… 13
Figure S23. COSY spectrum of **3**……… 14
Figure S24. HMBC spectrum of **3**……… 14
Figure S25. NOESY spectrum of **3**……… 15
Figure S26. ^{1}H NMR spectrum of **4** in $CDCl_3$ at 600 MHz……… 15
Figure S27. ^{13}C NMR spectrum of **4** in $CDCl_3$ at 150 MHz……… 16
Figure S28. ^{1}H NMR spectrum of **5** in $CDCl_3$ at 600 MHz……… 16
Figure S29. ^{13}C NMR spectrum of **5** in $CDCl_3$ at 150 MHz……… 17
Figure S30. ^{1}H NMR spectrum of **6** in $CDCl_3$ at 600 MHz……… 17
Figure S31. ^{13}C NMR spectrum of **6** in $CDCl_3$ at 150 MHz……… 18
Figure S32. ^{1}H NMR spectrum of **7** in $CDCl_3$ at 600 MHz……… 18
Figure S33. ^{13}C NMR spectrum of **7** in $CDCl_3$ at 150 MHz……… 19
Figure S34. Effects of capnellenes **1**–**7** on the protein expression levels of pro-inflammatory iNOS and COX-2 were assessed using immunoblot in RAW264.7 cell. 19

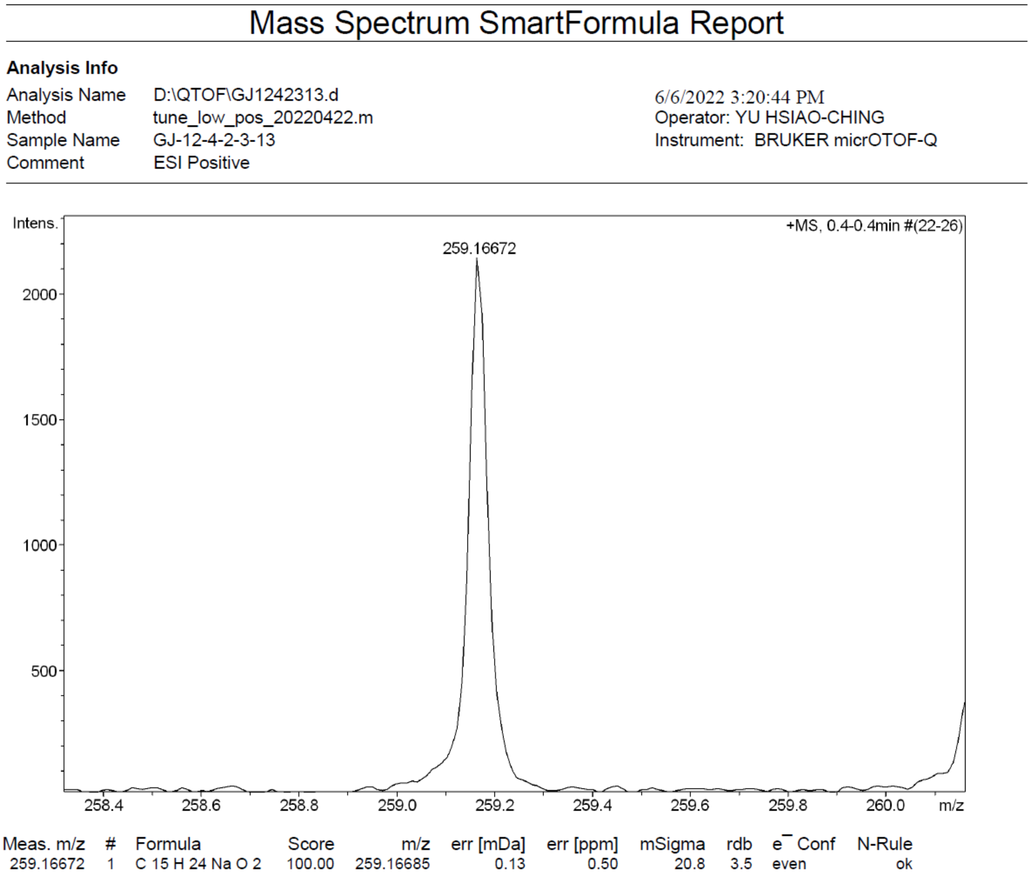

Figure S1. HRESIMS spectrum of **1**

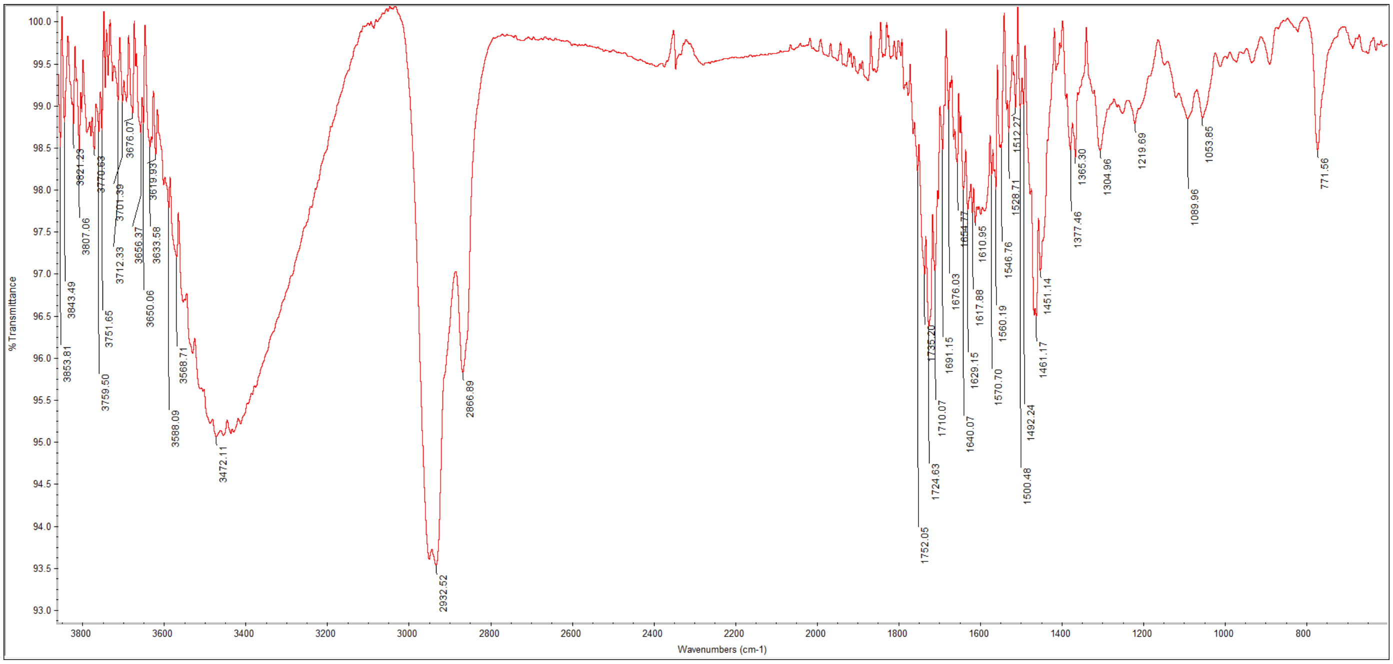

Figure S2. IR spectrum of **1**

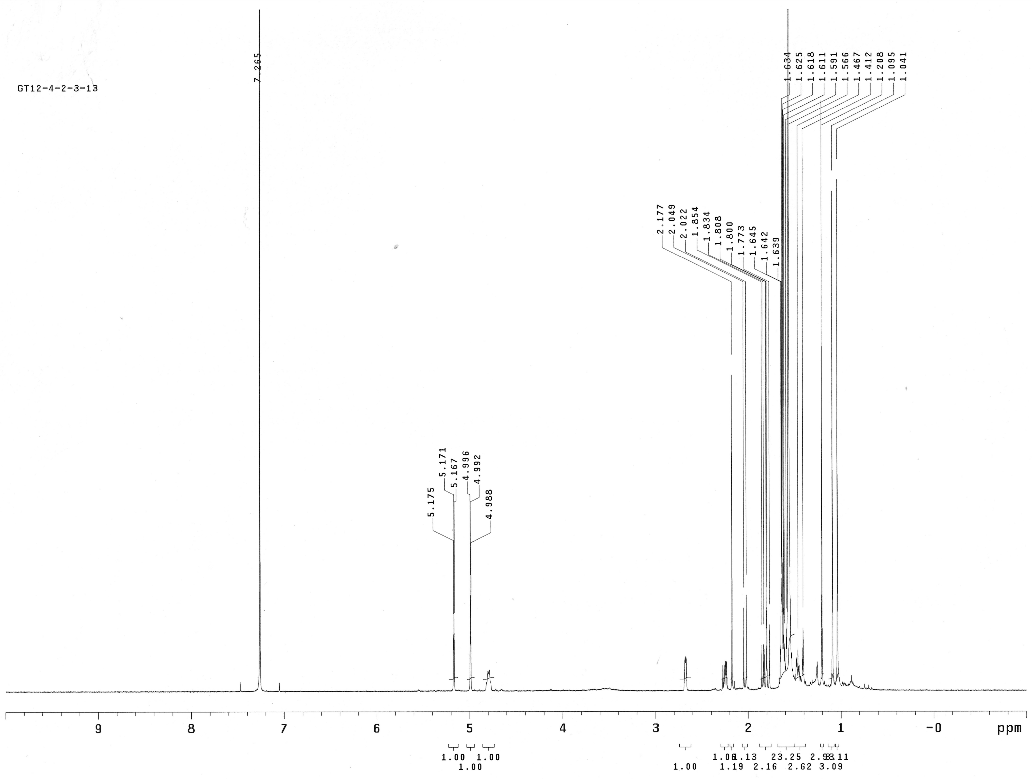

Figure S3. ^{1}H NMR spectrum of **1** in $CDCl_3$ at 500 MHz

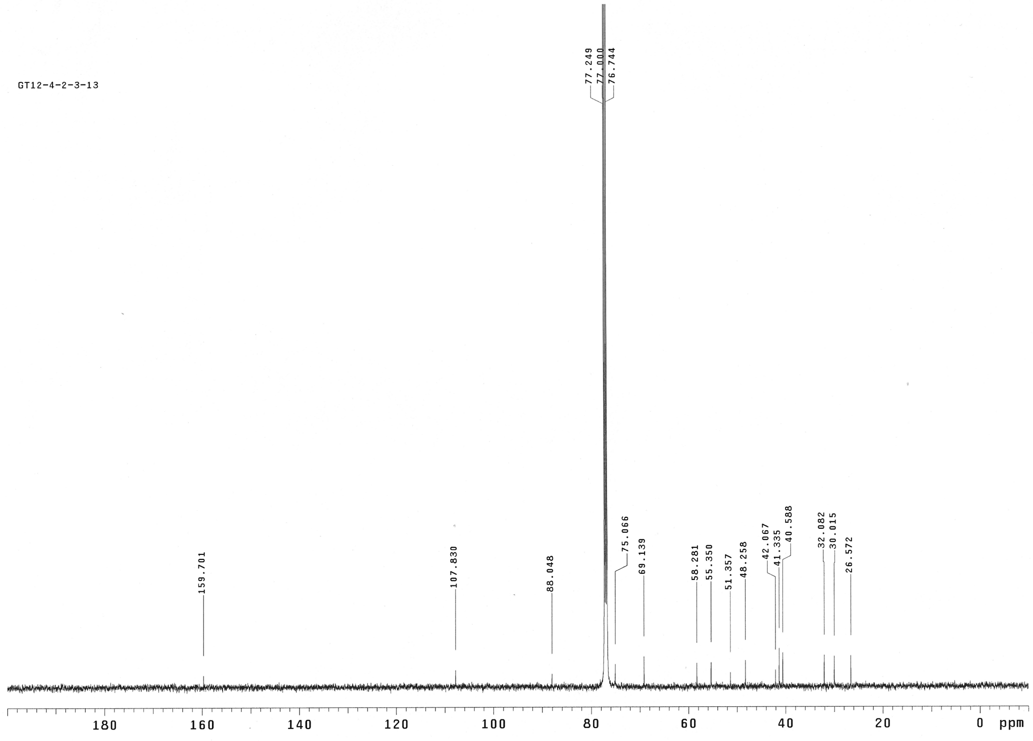

Figure S4. ^{13}C NMR spectrum of **1** in $CDCl_3$ at 125 MHz

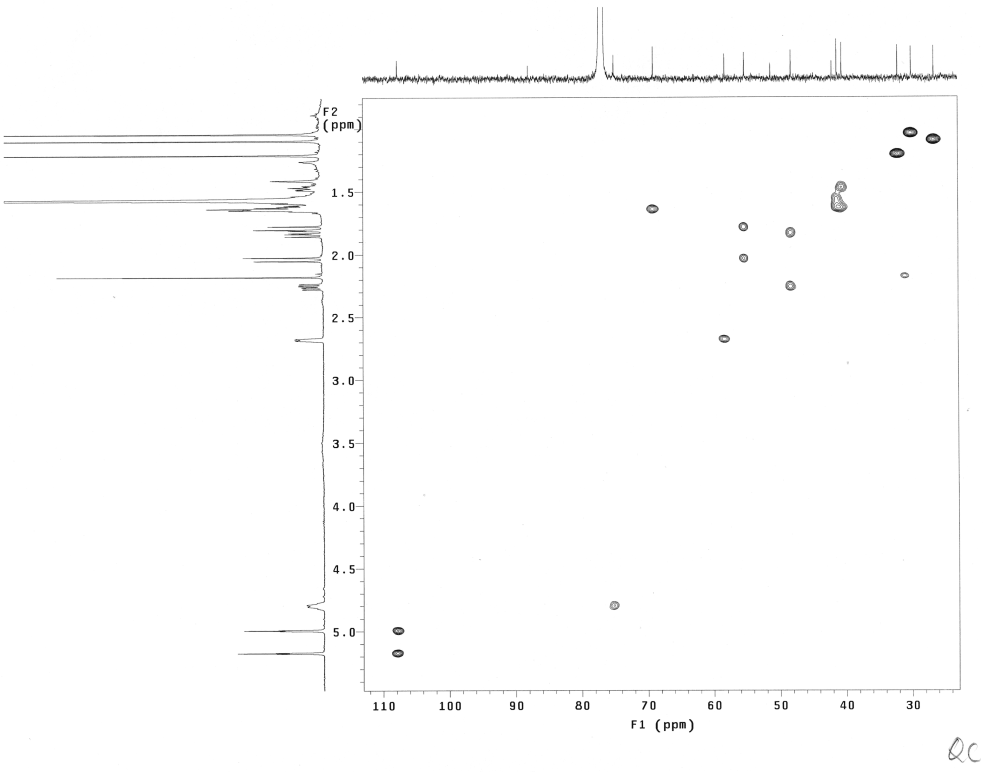

Figure S5. HMQC spectrum of **1**

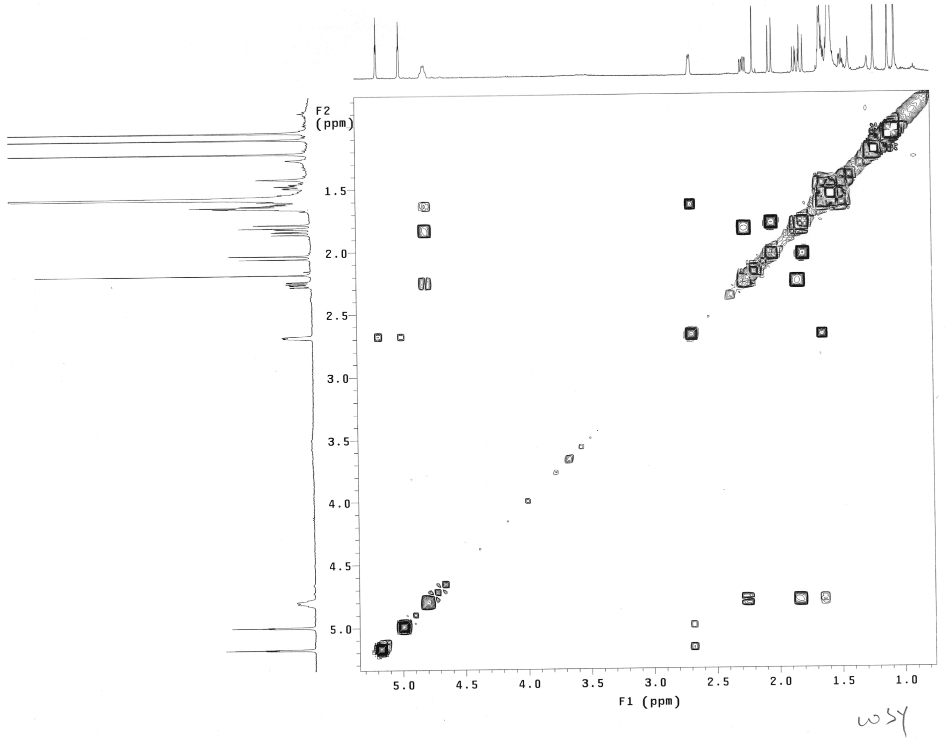

Figure S6. COSY spectrum of **1**

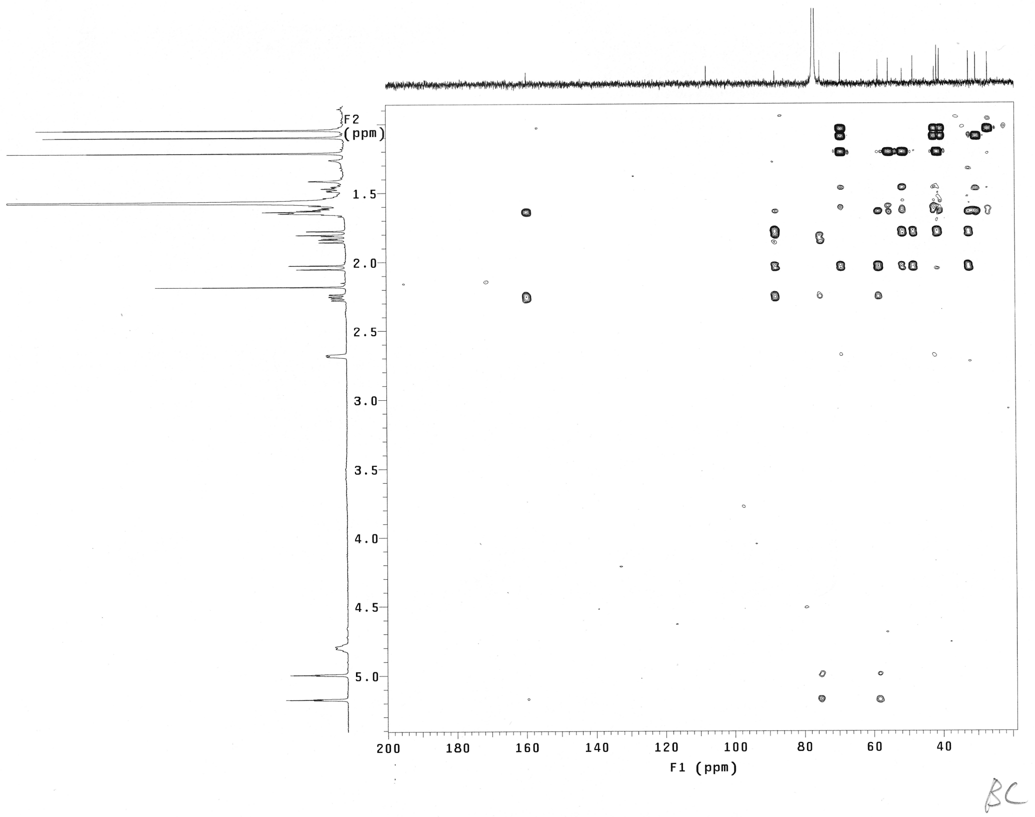

Figure S7. HMBC spectrum of **1**

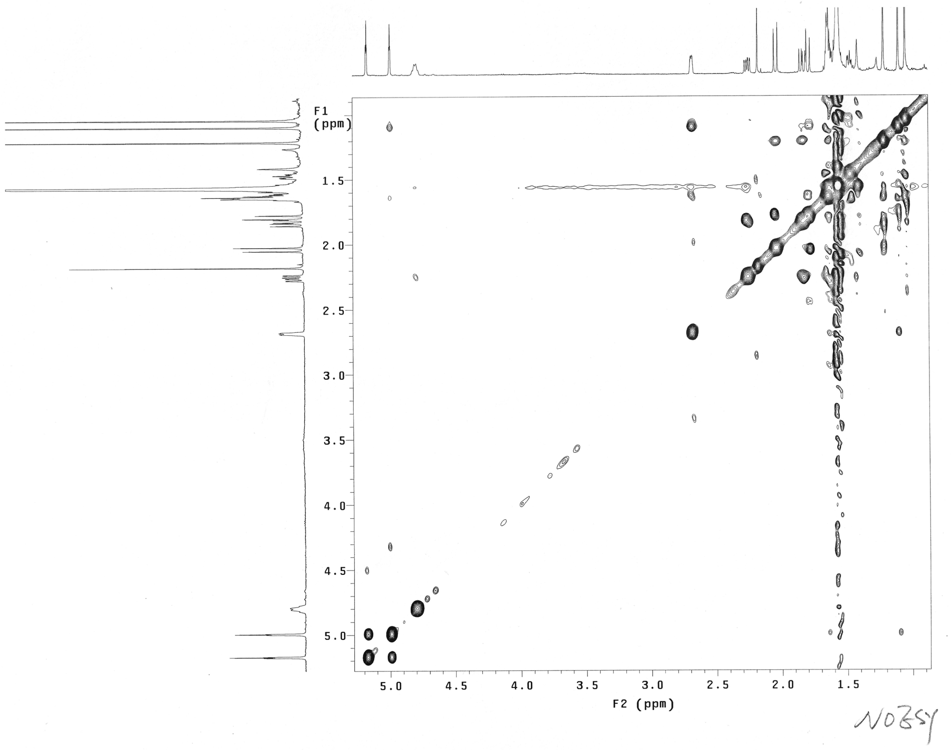

Figure S8. NOESY spectrum of **1**

Mass Spectrum SmartFormula Report

Analysis Info

Analysis Name	D:\QTOF\GJ1242311.d	5/31/2022 4:53:21 PM
Method	tune_low_pos_20220422.m	Operator: YU HSIAO-CHING
Sample Name	GJ-12-4-2-3-11	Instrument: BRUKER micrOTOF-Q
Comment	ESI Positive	

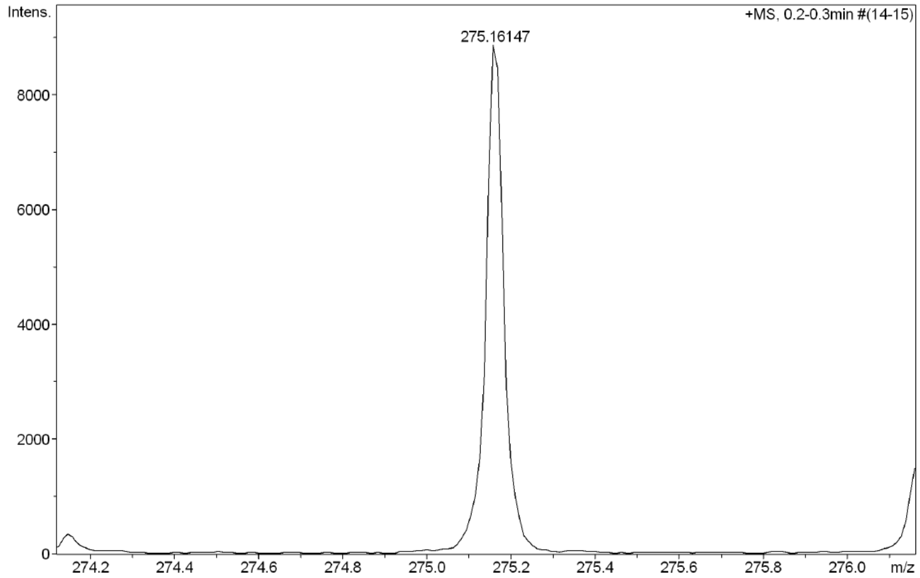

Meas. m/z	#	Formula	Score	m/z	err [mDa]	err [ppm]	mSigma	rdb	e⁻ Conf	N-Rule
275.16147	1	C 15 H 24 Na O 3	100.00	275.16177	0.29	1.06	28.7	3.5	even	ok

Figure S9. HRESIMS spectrum of **2**

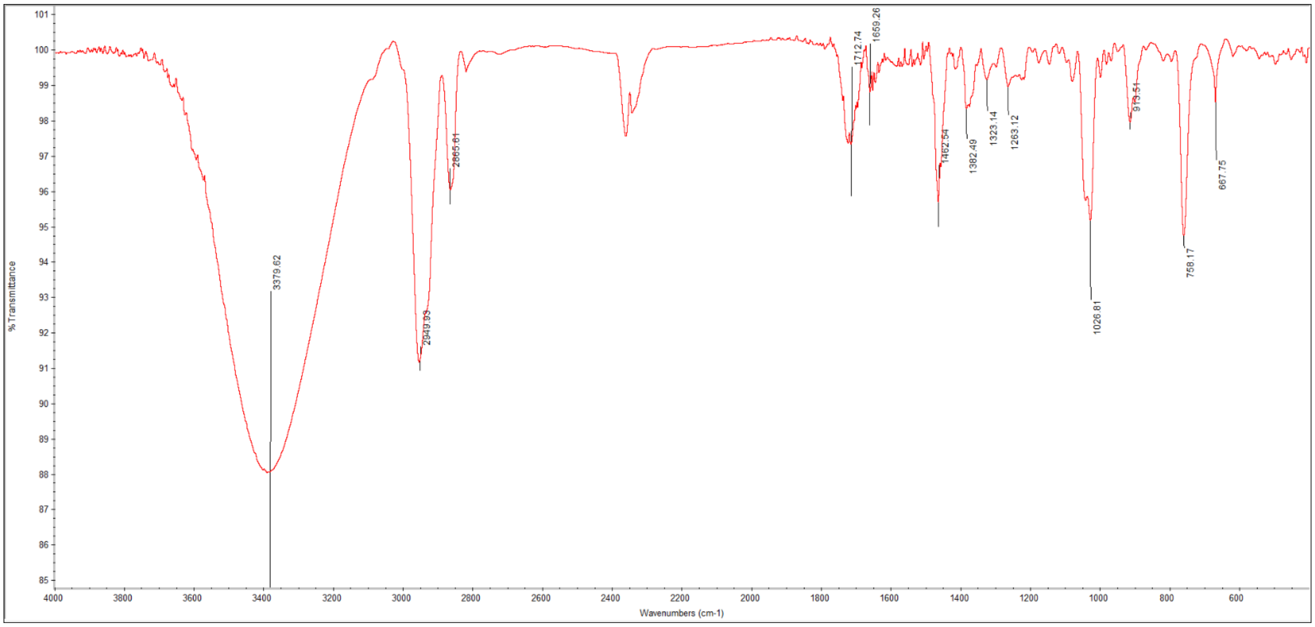

Figure S10. IR spectrum of **2**

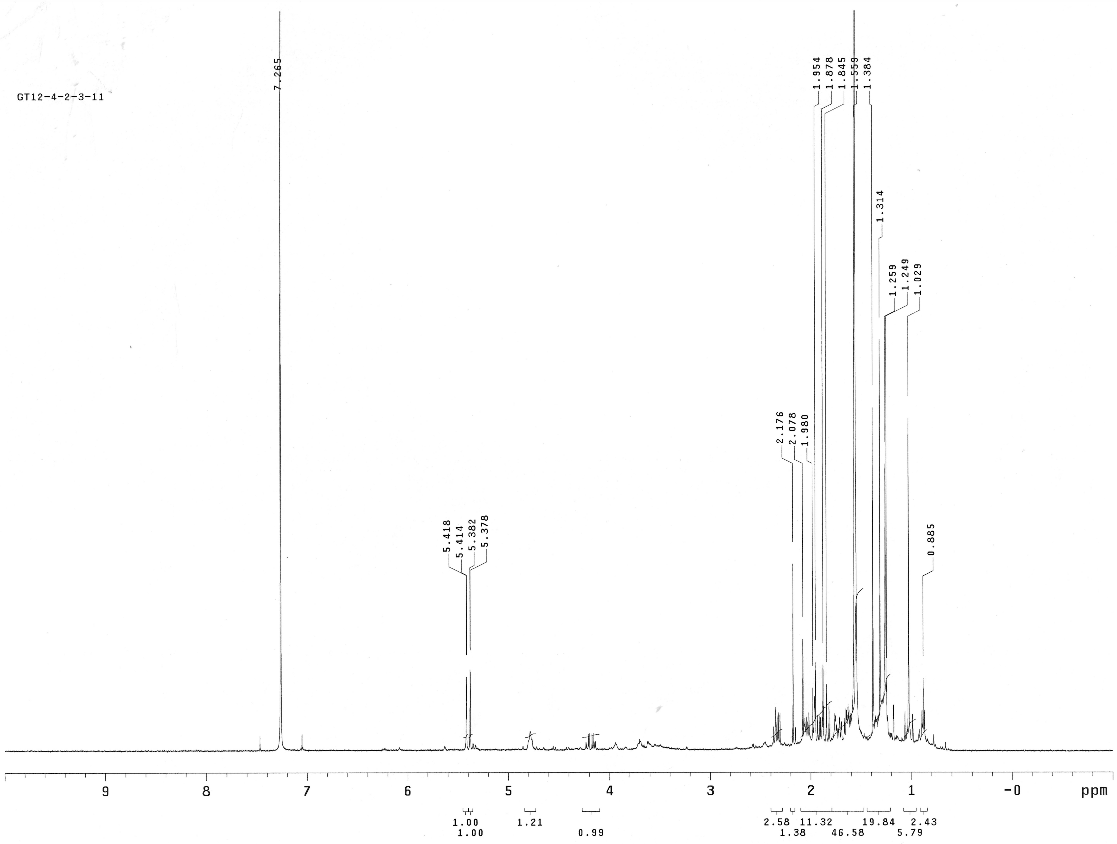

Figure S11. ^{1}H NMR spectrum of **2** in $CDCl_3$ at 500 MHz

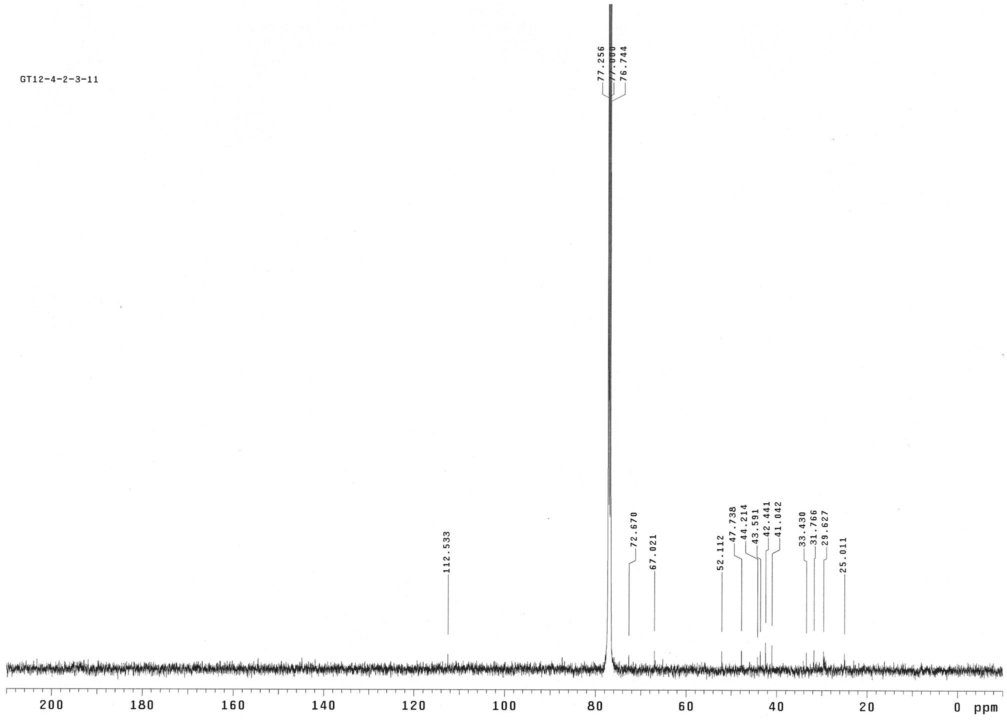

Figure S12. ^{13}C NMR spectrum of **2** in $CDCl_3$ at 125 MHz

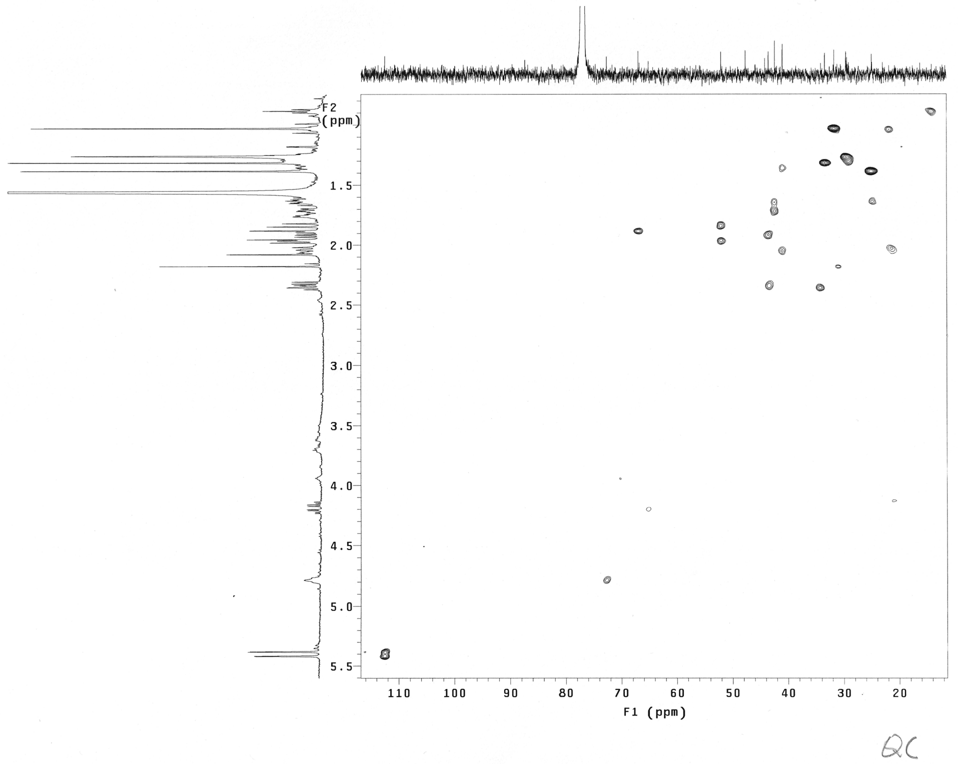

Figure S13. HMQC spectrum of **2**

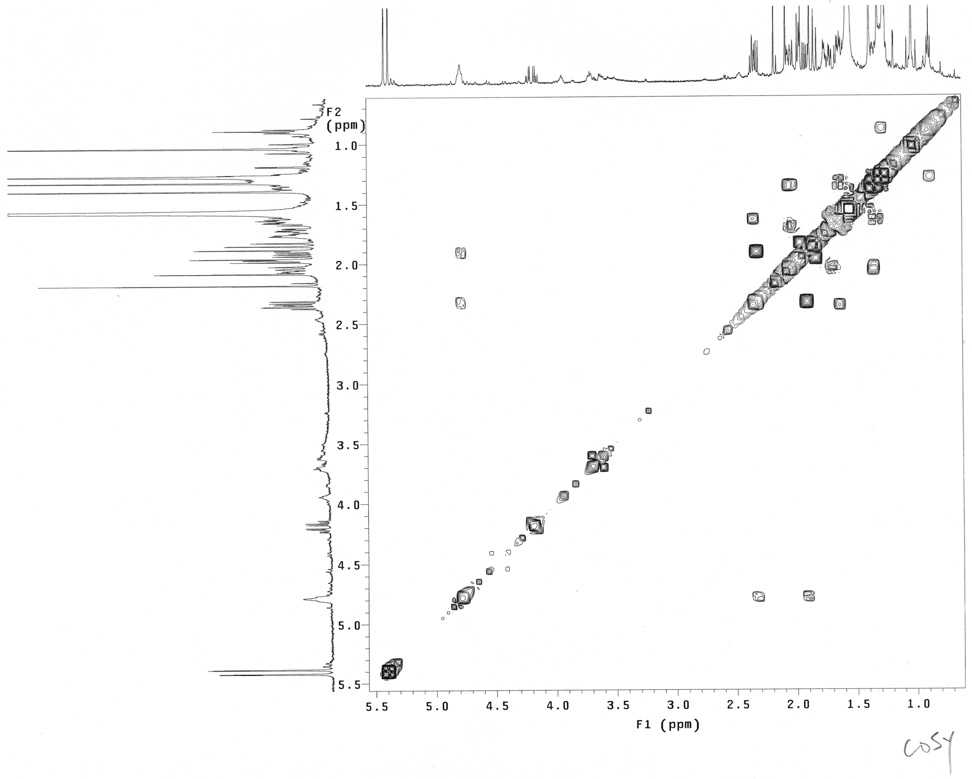

Figure S14. COSY spectrum of **2**

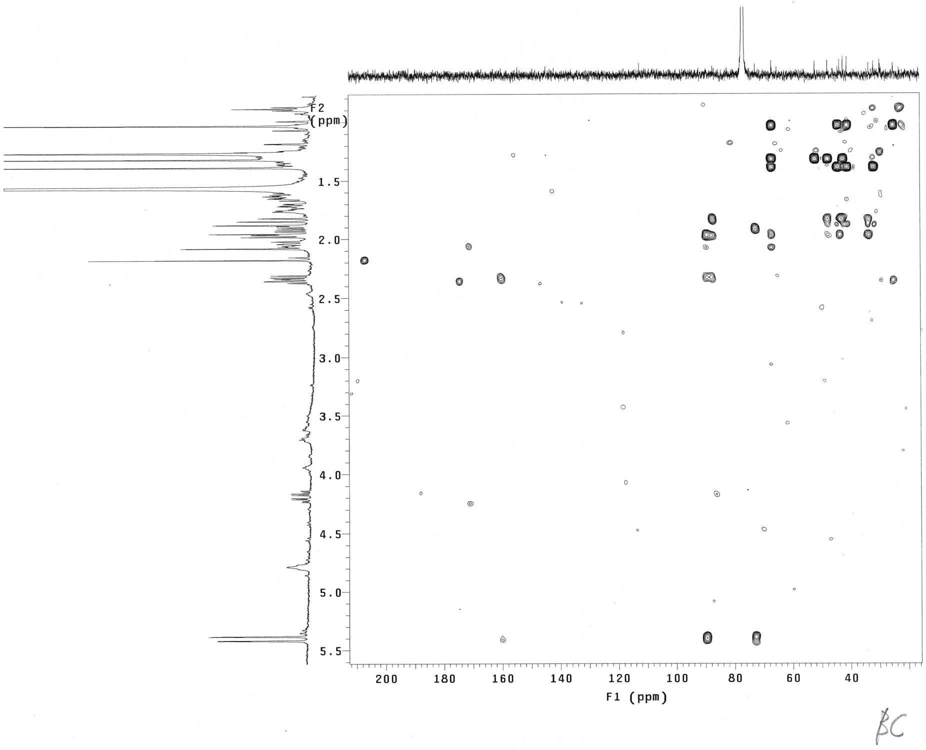

Figure S15. HMBC spectrum of **2**

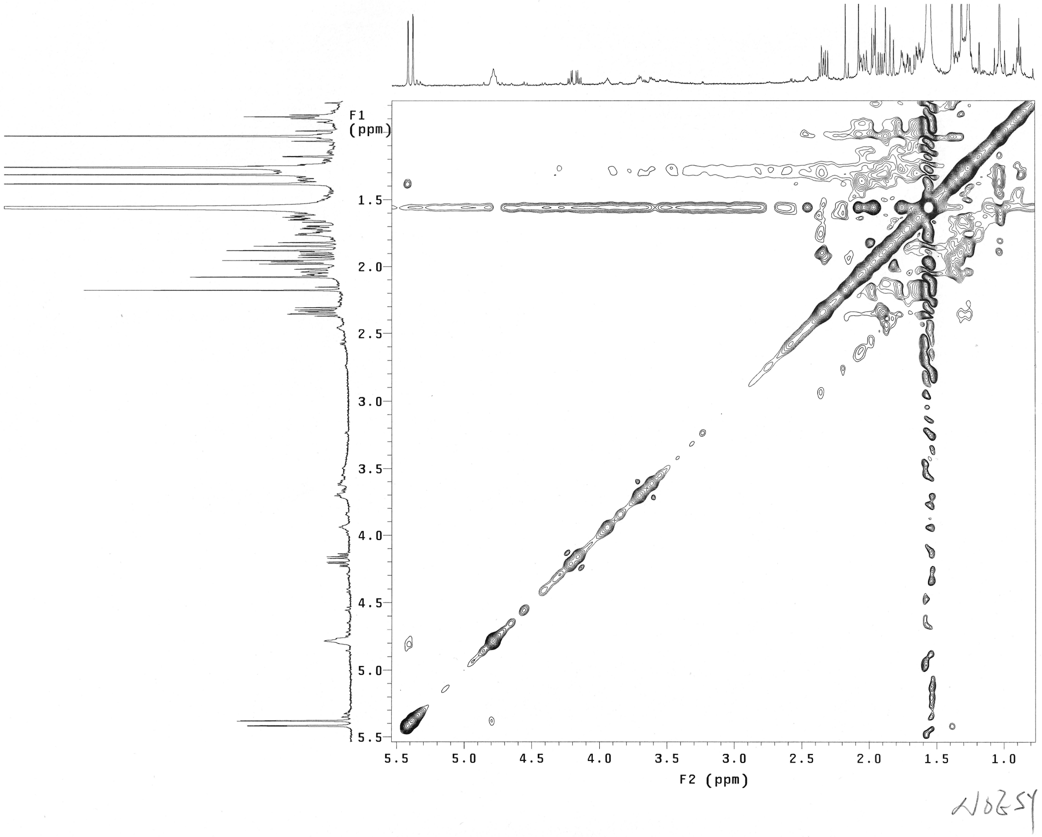

Figure S16. NOESY spectrum of **2**

Mass Spectrum SmartFormula Report

Analysis Info

Analysis Name	D:\QTOF\GJ138D3.d	7/1/2022 2:01:26 PM
Method	tune_low_pos_20220422.m	Operator: YU HSIAO-CHING
Sample Name	GJ-13-8	Instrument: BRUKER micrOTOF-Q
Comment	ESI Positive	

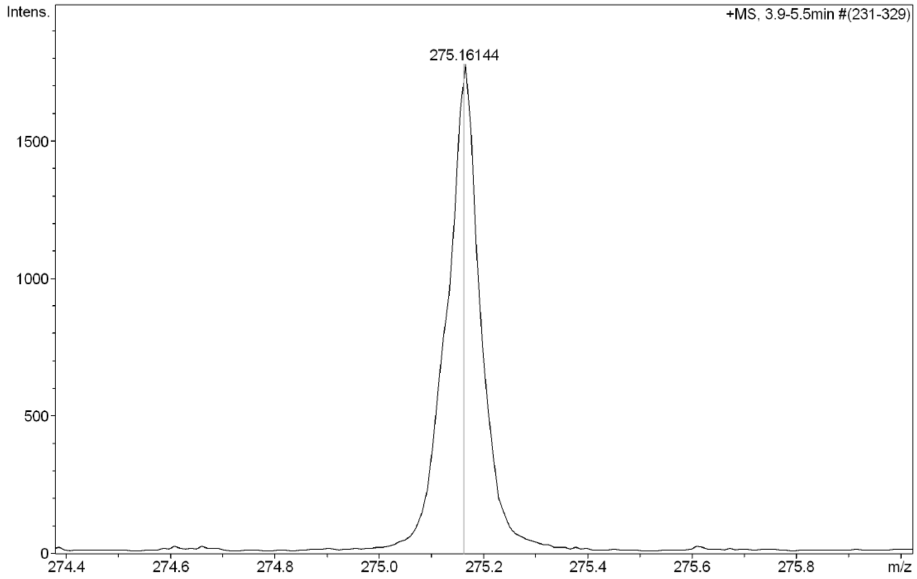

Meas. m/z	#	Formula	Score	m/z	err [mDa]	err [ppm]	mSigma	rdb	e⁻ Conf	N-Rule
275.16144	1	C 15 H 24 Na O 3	100.00	275.16177	0.33	1.19	211.5	3.5	even	ok

Figure S17. HRESIMS spectrum of **3**

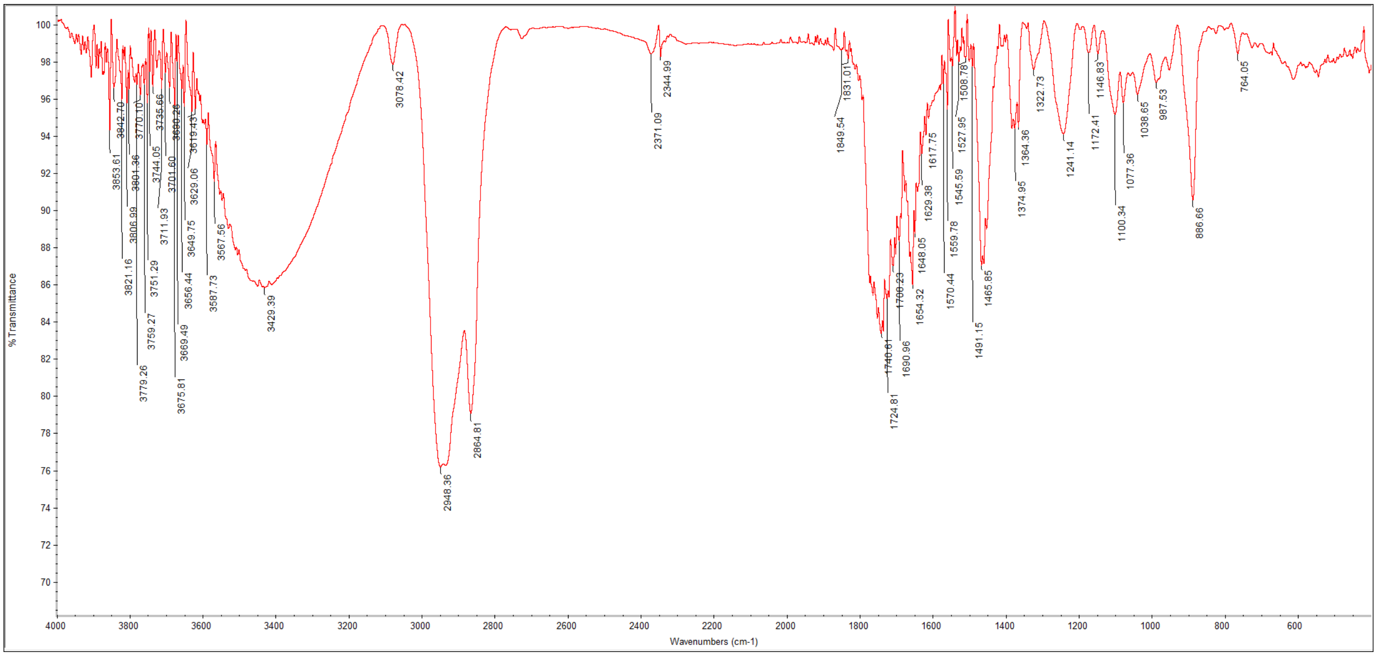

Figure S18. IR spectrum of **3**

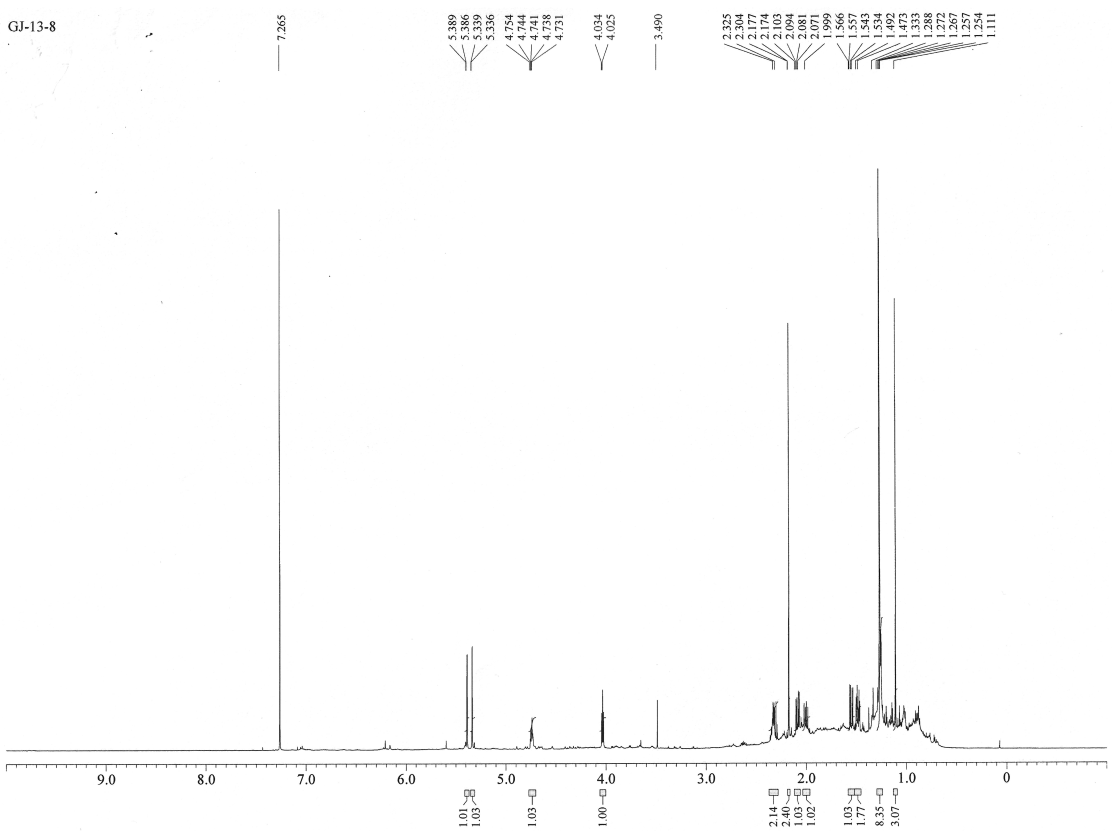

Figure S19. ^{1}H NMR spectrum of **3** in $CDCl_3$ at 600 MHz

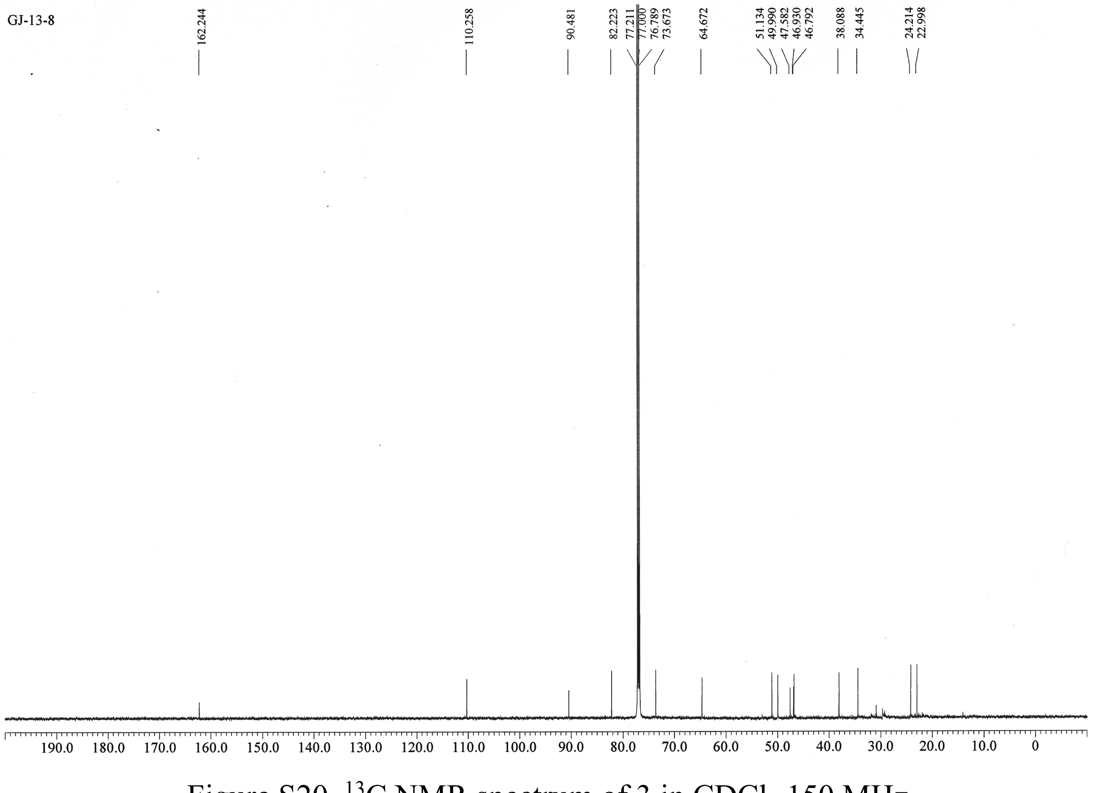

Figure S20. ^{13}C NMR spectrum of 3 in $CDCl_3$ 150 MHz

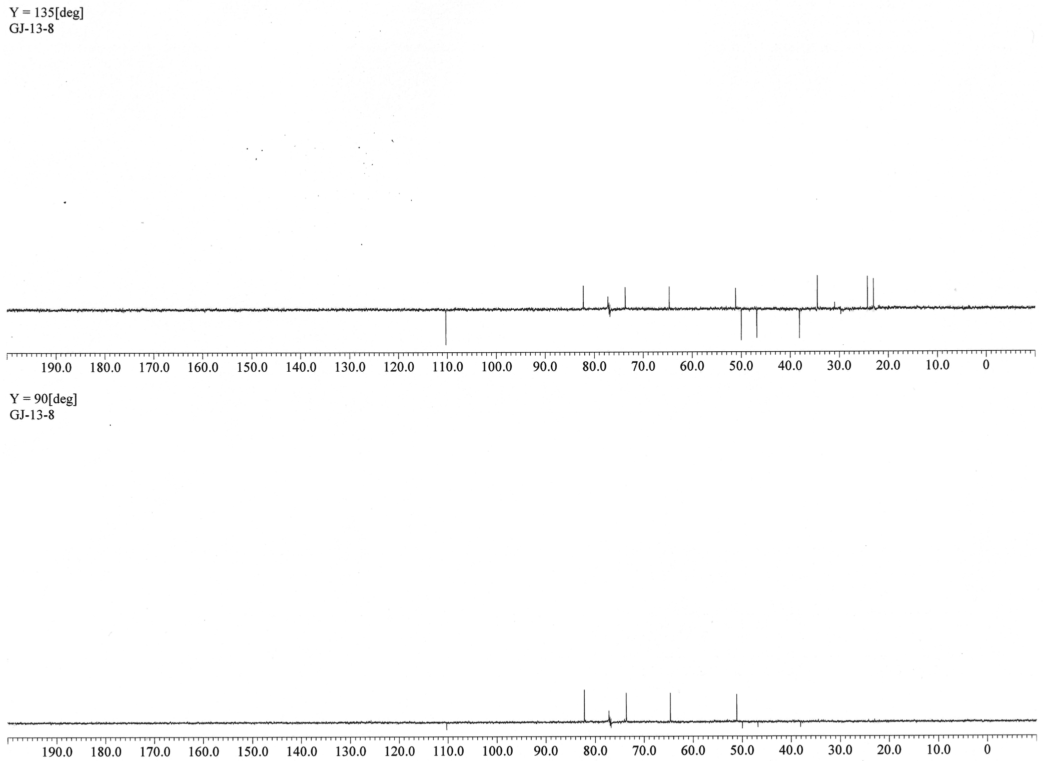

Figure S21. DEPT spectrum of **3**

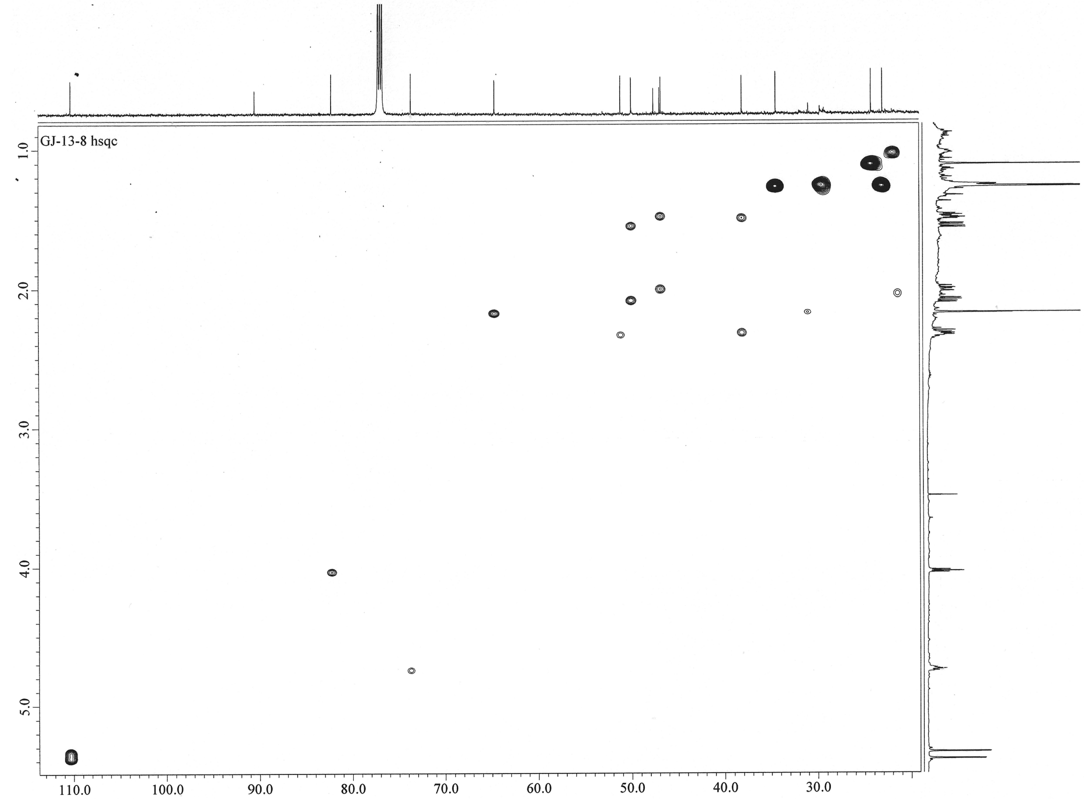

Figure S22. HMQC spectrum of **3**

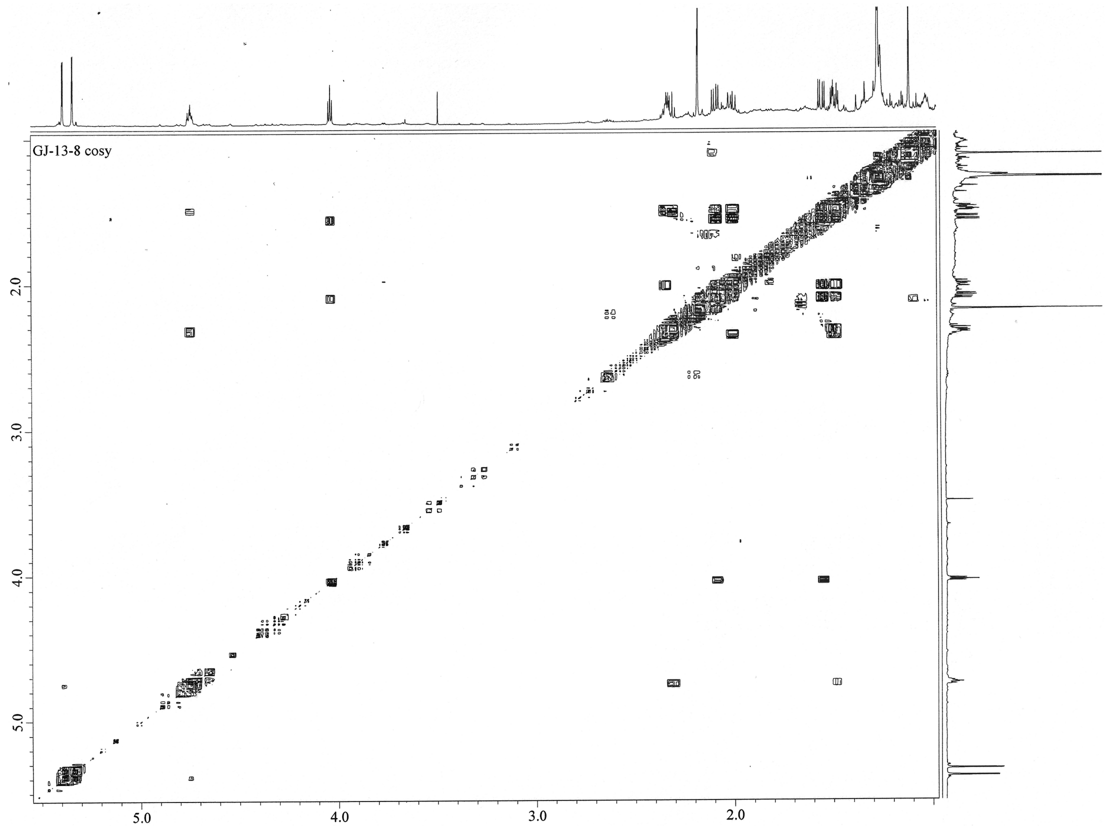

Figure S23. COSY spectrum of **3**

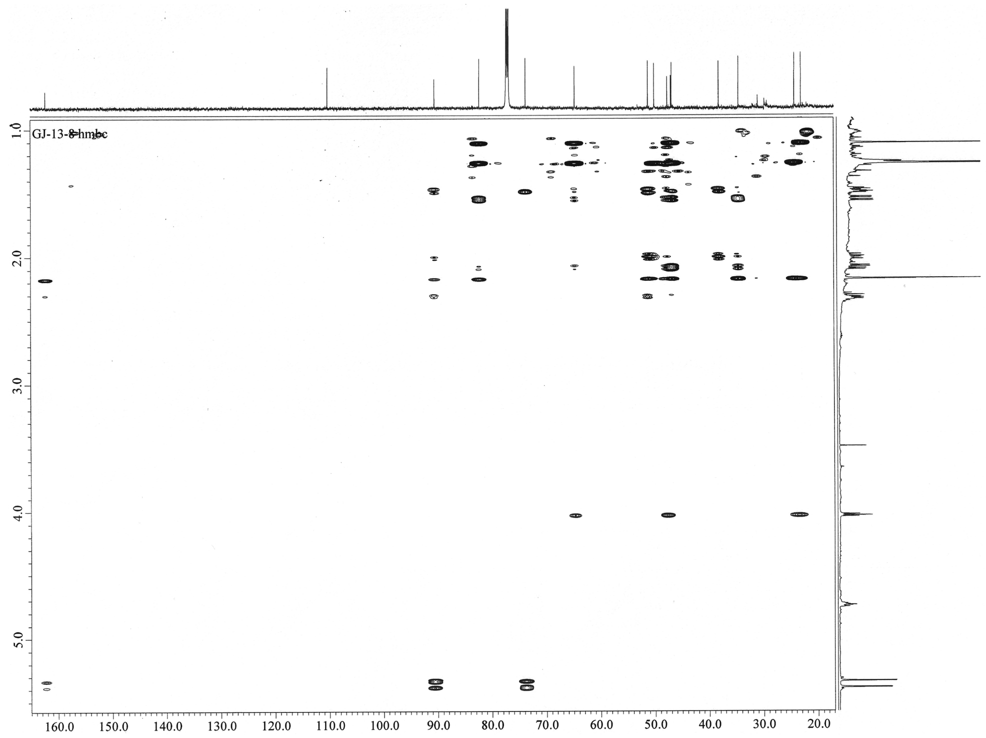

Figure S24. HMBC spectrum of **3**

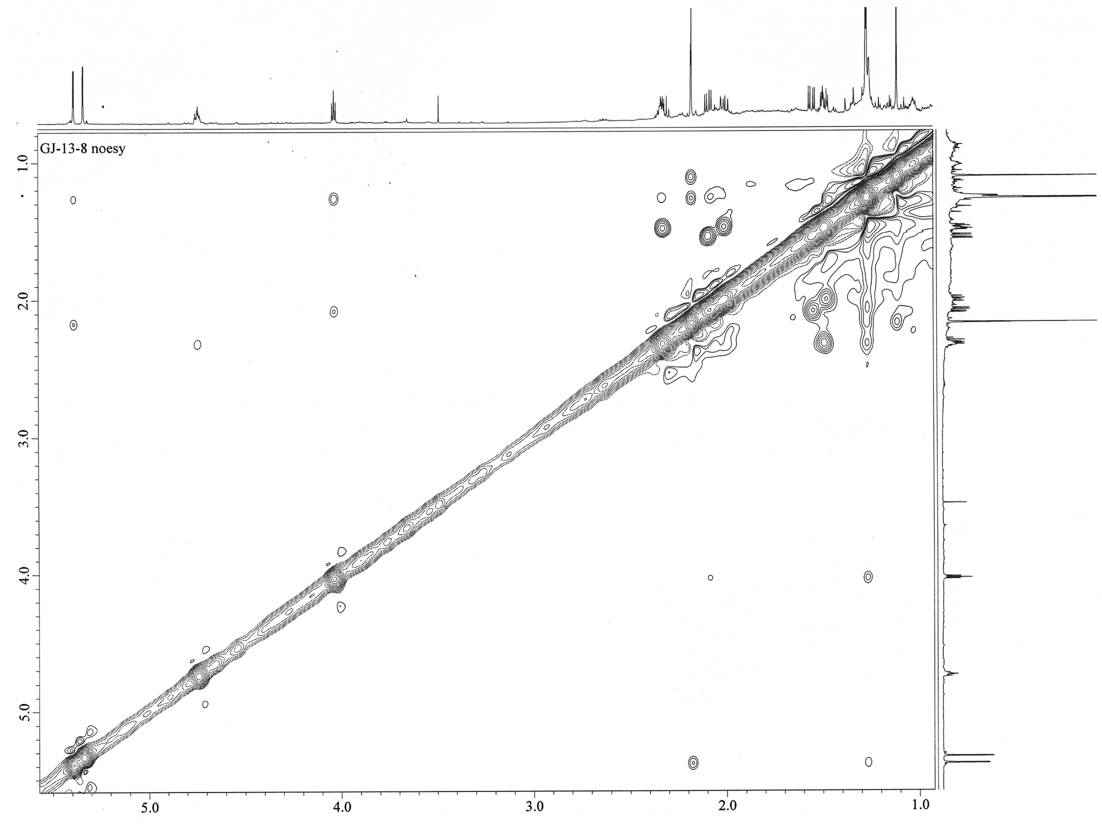

Figure S25. NOESY spectrum of **3**

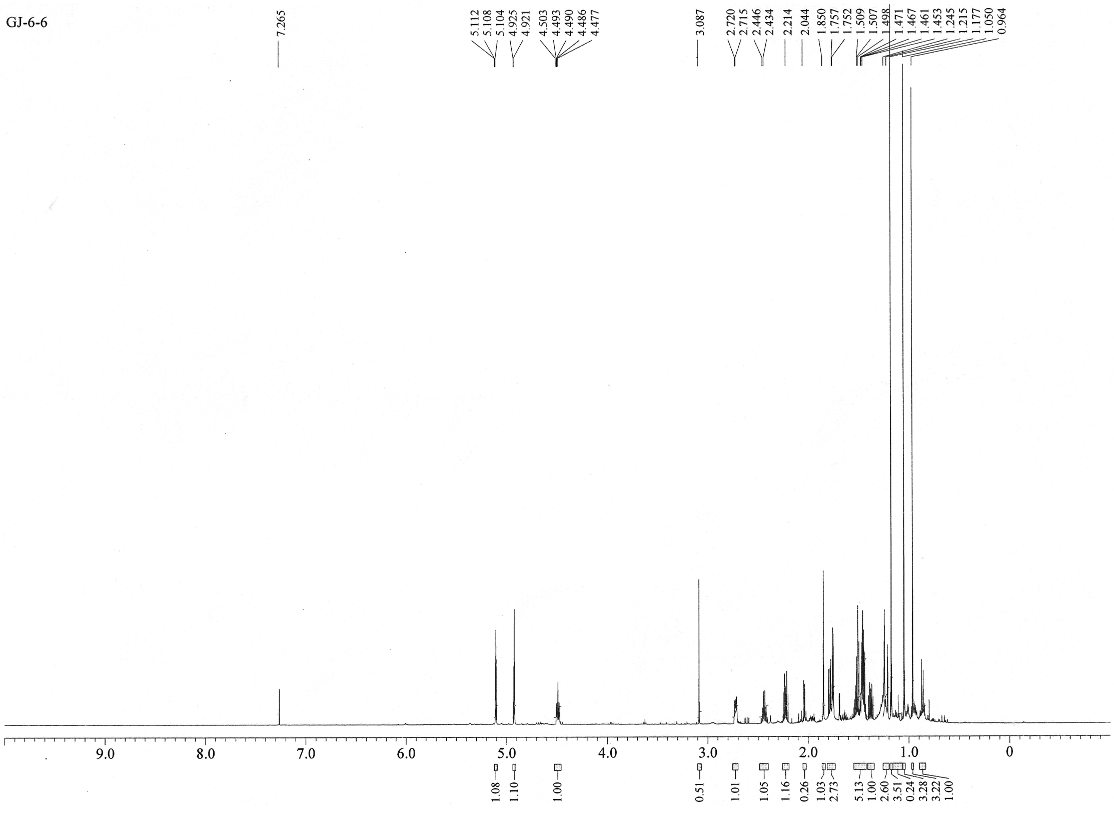

Figure S26. ^{1}H NMR spectrum of **4** in $CDCl_3$ at 600 MHz

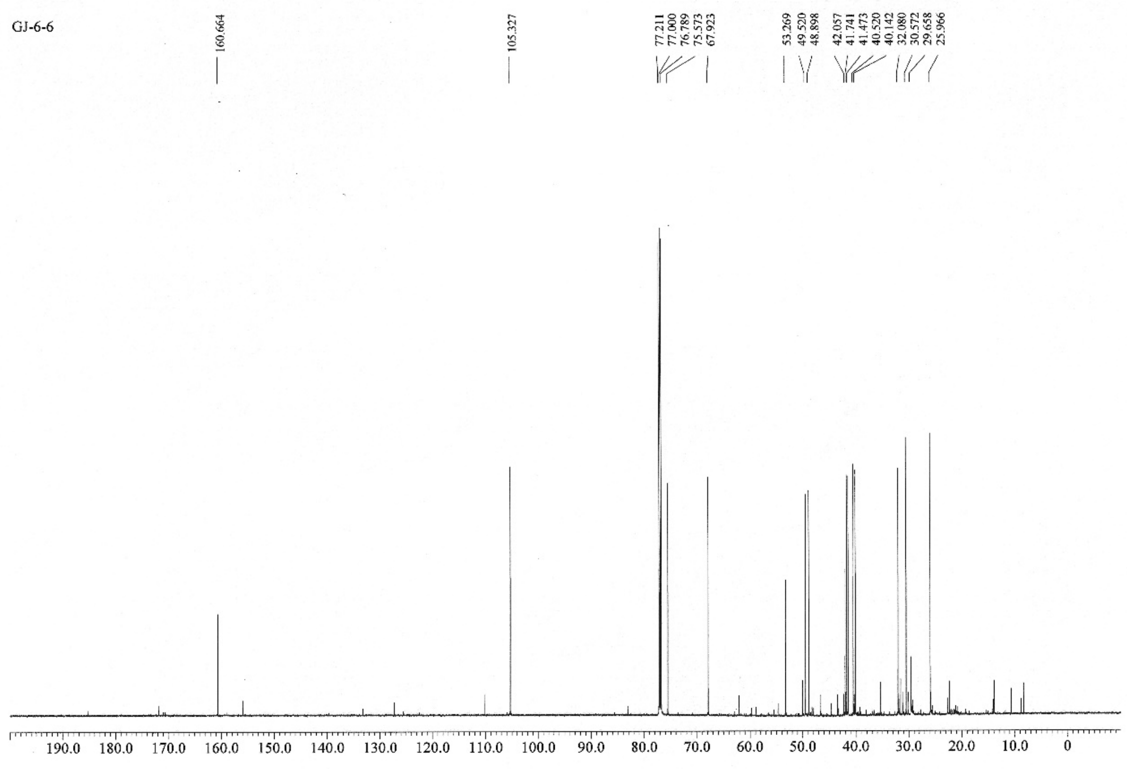

Figure S27. ^{13}C NMR spectrum of **4** in $CDCl_3$ 150 MHz

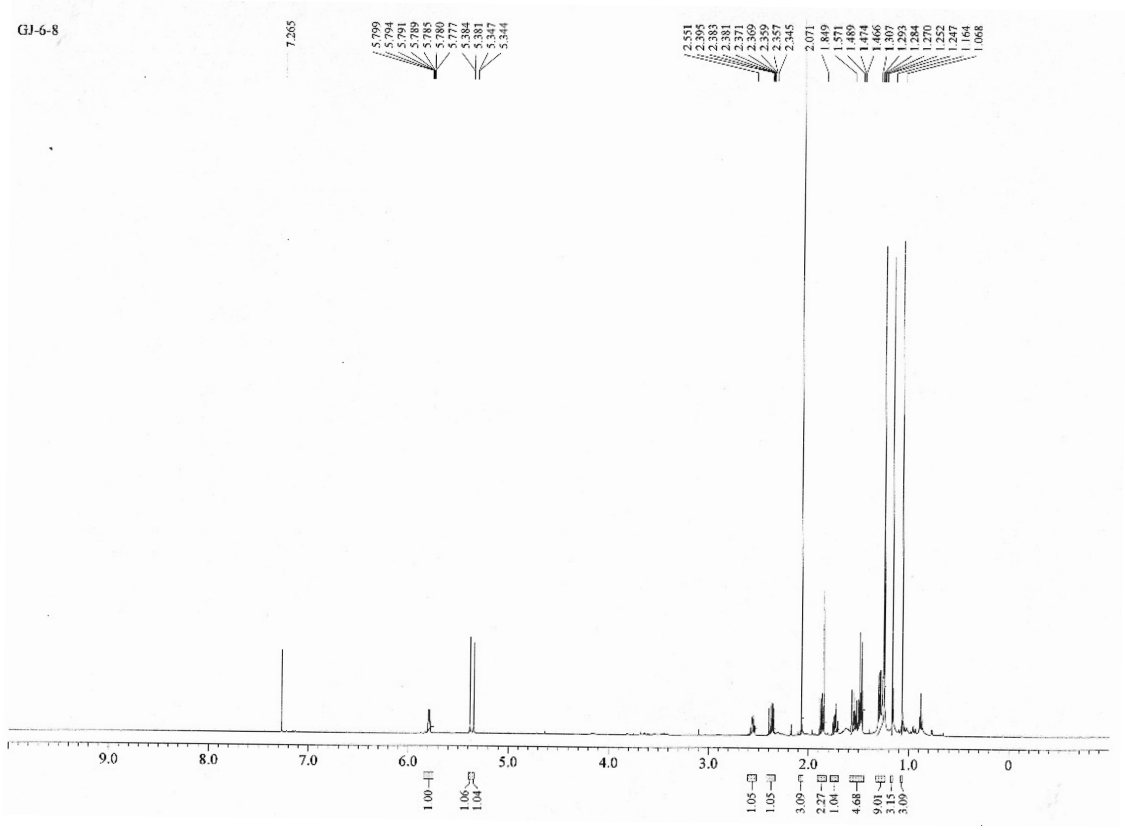

Figure S28. ^{1}H NMR spectrum of **5** in $CDCl_3$ at 600 MHz

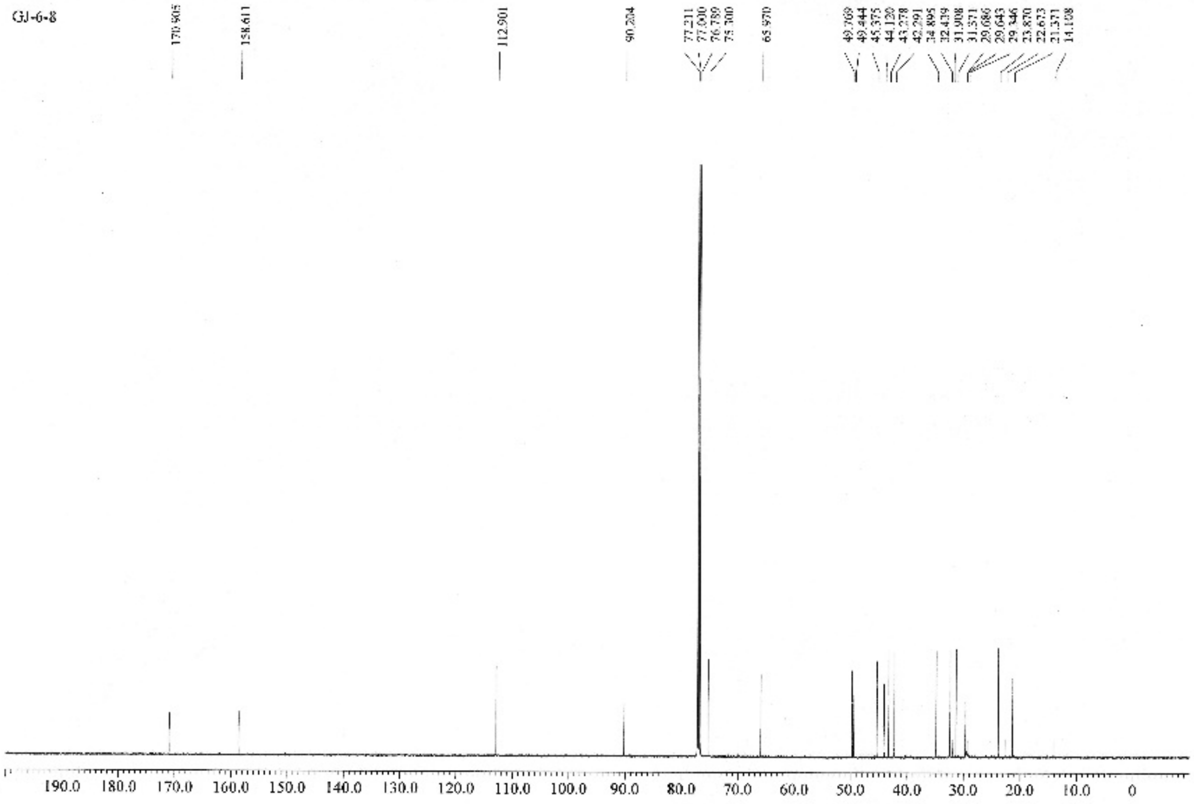

Figure S29. ^{13}C NMR spectrum of **5** in $CDCl_3$ 150 MHz

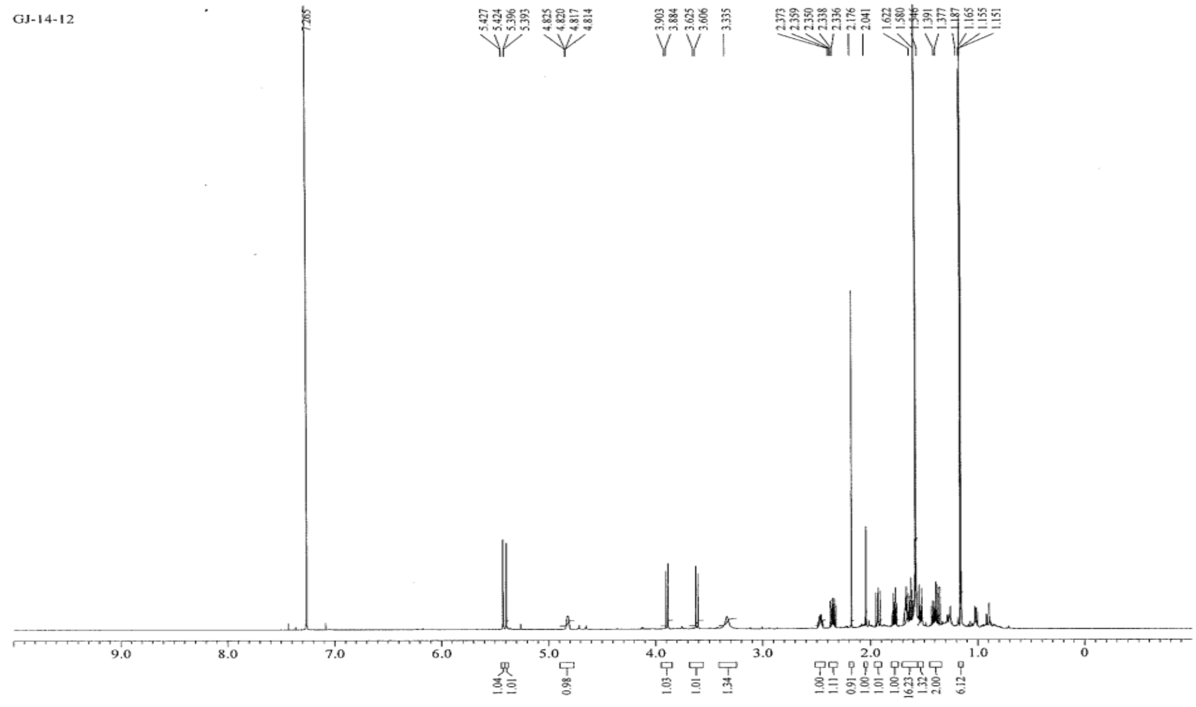

Figure S30. ^{1}H NMR spectrum of **6** in $CDCl_3$ at 600 MHz

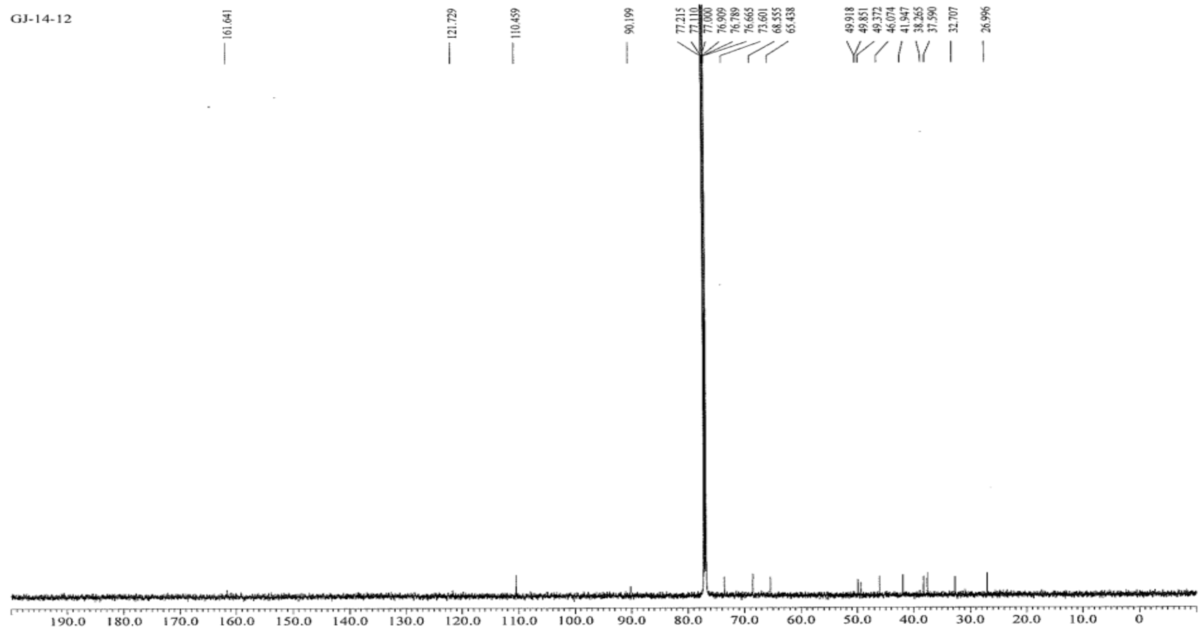

Figure S31. ^{13}C NMR spectrum of **6** in $CDCl_3$ at 150 MHz

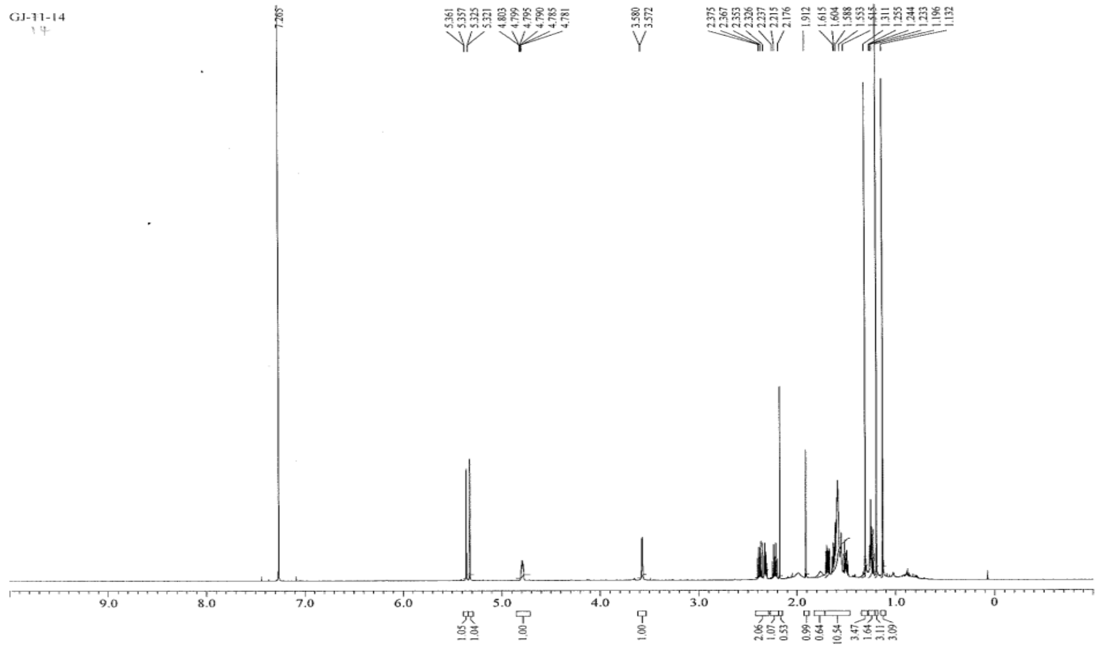

Figure S32. ^{1}H NMR spectrum of **7** in $CDCl_3$ at 600 MHz

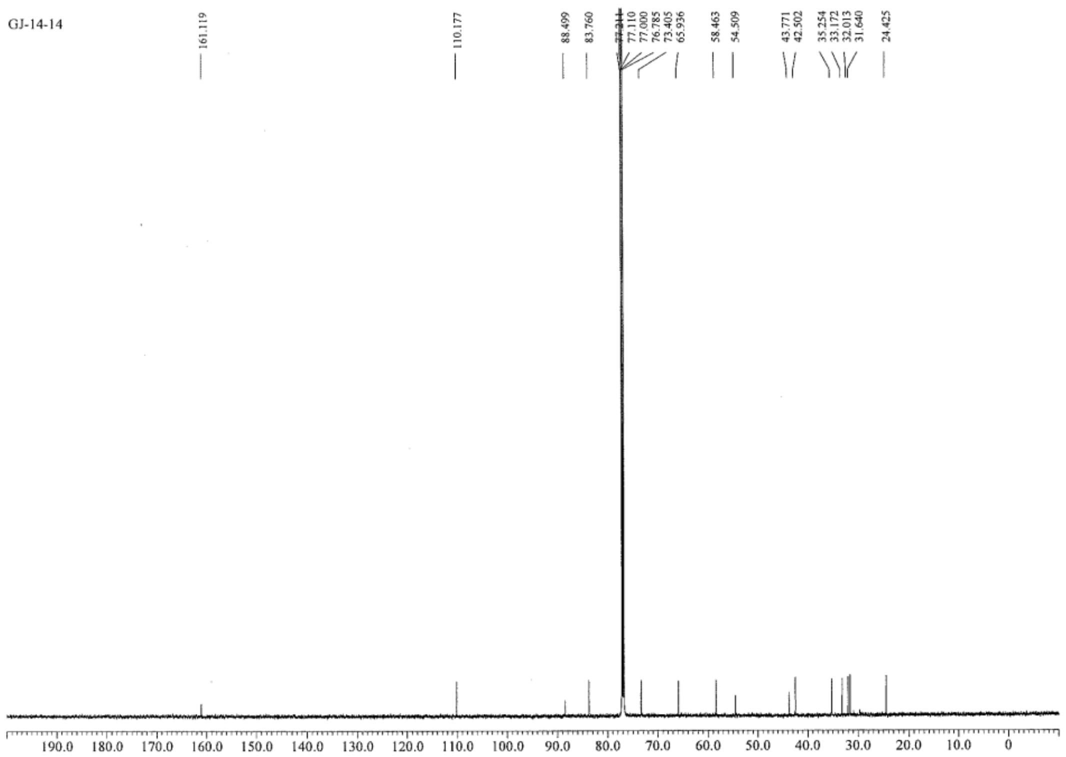

Figure S33. ^{13}C NMR spectrum of **7** in $CDCl_3$ at 150 MHz

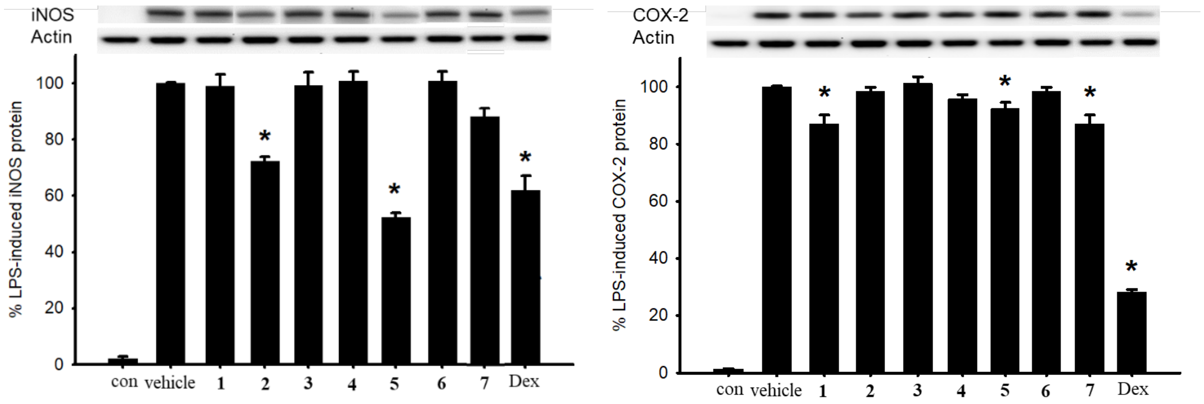

Figure S34. Effects of capnellenes **1**–**7** on the protein expression levels of pro-inflammatory iNOS and COX-2 were assessed using immunoblot in RAW264.7 cell. Data were standardized to the LPS-treated cells. The dexamethasone (Dex.) were used as positive control. Data are expressed as the mean ± SEM (n = 3). $^{*}p < 0.05$, compared with the group of LPS-treated cells.
